# Tumor-associated bacteria activate PRDX1-driven glycolysis to promote immune evasion and PD-1 antibody resistance in hepatocellular carcinoma

**DOI:** 10.3389/fmicb.2025.1599691

**Published:** 2025-07-07

**Authors:** Heng Zhang, Xi Lan, Liquan Cai, Xunfeng Gao, Feng Gao, Dan Yu, Jinlong Zhang, Jinhui Zhang, Qinwen Tai

**Affiliations:** ^1^General Surgery Center, Shenzhen Hospital, Southern Medical University, Shenzhen, China; ^2^Clinical Laboratory Center, Shenzhen Hospital, Southern Medical University, Shenzhen, China

**Keywords:** hepatocellular carcinoma, single-cell multiomics, bacterial infection, evasion, PD-1 antibody resistance, PRDX1/NF-κB signaling

## Abstract

**Background:**

Recent studies have highlighted the presence of intratumoral bacteria in hepatocellular carcinoma (HCC), yet their contribution to immunotherapy resistance remains largely unexplored. This study investigates the mechanisms by which bacterial infection reshapes tumor metabolism to undermine the efficacy of anti-PD-1 therapy.

**Methods:**

We conducted 16S rRNA gene sequencing on 29 HCC clinical samples and integrated the data with single-cell RNA sequencing of 12,487 cells to map microbial, metabolic, and immune interactions within the tumor microenvironment. Functional validation was performed using orthotopic HCC mouse models (*n* = 8 per group), coupled with flow cytometry-based immune profiling.

**Results:**

Enrichment of *Streptococcaceae* was strongly associated with upregulation of key glycolytic enzymes (LDHA, PKM2; *p* < 0.001) and dysfunction of natural killer cells (reduced CD56^dim^/CD16^bright^ populations; hazard ratio = 2.15, 95% CI: 1.34–3.42). Mechanistically, bacterial colonization induced peroxiredoxin 1 (PRDX1) expression via the NF-κB pathway. This led to excessive lactate production, which suppressed CD8^+^ T cell cytotoxicity (*p* = 0.003) and increased the expression of immune checkpoint molecules (TIM-3: 2.7-fold; LAG-3: 1.9-fold). *In vivo*, bacterial infection decreased the antitumor efficacy of PD-1 blockade by 43% (tumor volume vs. control; *p* = 0.008), an effect that was reversed upon PRDX1 inhibition.

**Conclusion:**

Our findings identify PRDX1 as a central node in bacteria-driven metabolic reprogramming that facilitates immune evasion and resistance to PD-1 therapy in HCC. These findings provide the first evidence linking intratumoral bacteria to PD-1 resistance via redox-regulated metabolism, proposing dual targeting of PRDX1 and gut microbiota as a novel combinatorial immunotherapy strategy.

## Graphical abstract

Regulation of bacterial-metabolite-gene interaction networks in the liver cancer microenvironment and their role in immune evasion and tumor progression.
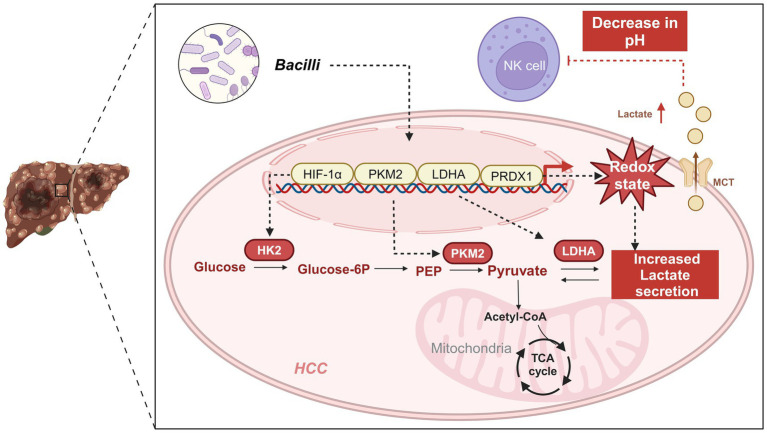

## Introduction

Hepatocellular carcinoma (HCC) is among the most prevalent and lethal malignancies worldwide, with persistently high incidence and mortality rates (Wang and Deng, 2023; Jiang et al., 2023; Ioannou, 2021). Although advances in treatment modalities—such as surgical resection, radiotherapy, chemotherapy, and targeted therapy—have improved clinical outcomes, long-term survival in HCC remains unsatisfactory. In recent years, immunotherapy has emerged as a promising strategy for HCC by mitigating pro-tumor immune effects or enhancing anti-tumor immunity. Within the unique immune microenvironment of HCC, targeting immune cells can induce diverse functional changes, making the tumor immune microenvironment (TIME) an attractive therapeutic target (Pan et al., 2024). Immune checkpoint inhibitors, in particular, have shown clinical benefit in some patients (Chen et al., 2024; Sirera et al., 2025; Pfister et al., 2021); however, a substantial proportion of patients still fail to respond significantly (Ji et al., 2024). The limited efficacy of immunotherapy is thought to be closely associated with the regulatory influence of non-tumor cells within the tumor microenvironment (TME) (Lee et al., 2024; Di Marco et al., 2025). This microenvironment consists of a complex interplay among various cellular components, extracellular matrix elements, and cytokines, collectively modulating tumor progression and treatment responses (Liu H. et al., 2024; Liu P. et al., 2024).

Among these non-tumor elements, tumor-associated bacteria have garnered increasing attention for their roles in modulating tumor biology (Peng et al., 2022). These microbial communities, residing within the tumor or originating from the gut, can alter host metabolism and gene regulation (Guan et al., 2024a, 2024b), thereby contributing to HCC development and progression. Recent studies have reported that bacterial communities residing in the gut and tumor tissues can modulate host immune responses and metabolic pathways, thereby affecting tumor growth and treatment efficacy (Zhou et al., 2024; Galeano Niño et al., 2022). For example, certain bacteria promote tumor cell survival and proliferation by altering local pH or producing specific metabolic byproducts. Moreover, bacteria can modulate the host immune system (Du et al., 2025), including through the regulation of cytokine and chemokine expression within the TME (Yang C. et al., 2024; Yang L. et al., 2024), thereby affecting immune cell recruitment and activation (Sun et al., 2024). These findings underscore the intricate interactions between bacteria and tumor cells and suggest novel therapeutic targets (Koelsch et al., 2024).

Peroxiredoxin 1 (PRDX1), a redox-regulating antioxidant enzyme, has been implicated in various cancer-related processes (Guan et al., 2024a, 2024b; Song et al., 2024; Min et al., 2018). In particular, PRDX1 plays pivotal roles in maintaining redox homeostasis and modulating key signaling pathways (Sun et al., 2023). In HCC, elevated PRDX1 expression is associated with enhanced glycolytic activity. Glycolysis, a hallmark metabolic pathway in cancer, serves as the principal source of energy and biosynthetic precursors necessary for rapid tumor proliferation (Kang et al., 2020; Li et al., 2022; Wang et al., 2022a, 2022b). Furthermore, increased glycolysis leads to the acidification of the TME, which in turn impairs immune cell function and facilitates immune evasion (Bader et al., 2024; Chang et al., 2015).

In this study, we sought to investigate the regulatory role of gut microbiota in HCC glycolysis, with a particular focus on PRDX1 and its impact on immunotherapy outcomes. By integrating single-cell transcriptomics and functional genomics analyses, we compared the microbial compositions of HCC patients and healthy individuals, and examined how bacterial differences influence tumor glycolytic pathways and the immune microenvironment. Our findings reveal that PRDX1 is a key mediator of bacteria-induced glycolysis in HCC and is closely associated with patient survival and immune cell infiltration. These results provide critical insights into how microbiota-driven metabolic reprogramming shapes the immunological landscape of HCC, highlighting PRDX1 as a potential therapeutic target. This study offers a scientific rationale for novel combination strategies aimed at improving immunotherapy efficacy in HCC by disrupting the bacteria-metabolism-immune axis, with substantial implications for both research and clinical practice.

## Materials and methods

### 16S rRNA sequencing data acquisition

Relevant datasets were identified in the EMBL-EBI database^1^ using the keyword “Liver cancer.” Phenotypic information for all samples within the selected project (BioProject ID: PRJNA1127013) was retrieved. The study included 17 HCC samples (tumors graded as stage III or above) and 12 healthy control samples. Corresponding 16S rRNA sequencing data were downloaded from the NCBI Sequence Read Archive (SRA) database.^2^

### Microbial relative abundance analysis

Sample quality was assessed using MultiQC (Ewels et al., 2016) and KneadData.^3^ MultiQC provided sequencing quality control metrics, while KneadData was used to filter out host-derived and contaminant sequences. Taxonomic profiles and phylogenetic relationships among microbial species were visualized and annotated using GraPhlAn (Bai et al., 2015), allowing the determination of microbial relative abundance. Alpha diversity (within-sample diversity) was evaluated using the Inverse Simpson index, while beta diversity (between-sample diversity) was assessed through principal coordinate analysis (PCoA). Statistical comparisons of microbial diversity and abundance were conducted using Wilcoxon rank-sum tests and Welch’s *t*-tests. Differential abundance analysis between groups was performed using the edgeR package in R, and the results were visualized using volcano plots and Manhattan plots. To further identify significantly different taxa, we applied the Linear Discriminant Analysis Effect Size (LEfSe) method (Erawijantari et al., 2020), with an LDA score cutoff of 2.0. Taxa with higher LDA scores indicated stronger group-specific enrichment. Significant findings were illustrated using bar plots that summarized differential taxonomic abundance.

### Microbial functional composition

Functional predictions of microbial communities were performed using the FAPROTAX database and software,^4^ a manually curated taxonomy-function mapping tool for prokaryotes. Data pre-processed via QIIME were converted using R packages and further analyzed with PICRUSt, which infers metagenomic functional content based on community phylogeny. The Kyoto Encyclopedia of Genes and Genomes (KEGG) database was utilized to predict functional pathways corresponding to each primer set. Statistical analyses and visualization of unstratified results were conducted using STAMP (v2.1.3), and Welch’s *t*-test was used to compare functional differences between groups.

### Data acquisition and processing

Single-cell RNA sequencing (scRNA-seq) data for HCC were obtained from the publicly available dataset GSE189903. This dataset includes tumor samples from four HCC patients (GSM5709317, GSM5709326, GSM5709327, and GSM5709336) and adjacent non-tumor liver tissue samples from three patients (GSM5709316, GSM5709324, and GSM5709329). Data processing was performed using the Seurat package in R. Quality control criteria were set as follows: 200 < nFeature_RNA < 5,000 and percent.mt < 20. The top 2,000 most variable genes, determined by variance, were selected for downstream analyses.

All datasets used in this study were obtained from publicly accessible databases. Therefore, ethical approval and informed consent were not required.

### Single-cell transcriptomic analysis

To reduce the dimensionality of the scRNA-seq dataset, principal component analysis (PCA) was performed based on the top 2,000 highly variable genes identified by variance. The first 20 principal components (PCs) were selected for downstream analysis, as determined by the ElbowPlot function in the Seurat package. Major cell subpopulations were identified using the FindClusters function (resolution = 1, default setting) in Seurat, followed by nonlinear dimensionality reduction via the Uniform Manifold Approximation and Projection (UMAP) algorithm. Marker genes for distinct cell populations were identified using Seurat’s built-in functions. Cell types were annotated by integrating known lineage-specific markers with references from the CellMarker database^5^ and the SingleR package in R. Intercellular communication networks were inferred using the CellChat R package.

Differentially expressed genes (DEGs) were identified using the Limma package in R. Genes were considered differentially expressed if they met the criteria of |log_2_ fold change| >0.5 and *p* < 0.05.

### Gene Ontology and KEGG enrichment analysis

Functional enrichment analysis was performed to explore the biological relevance of DEGs. Genes were first mapped to Gene Ontology (GO) terms using the org.Hs.eg.db annotation package (version 3.1.0). Enrichment analysis was carried out with the clusterProfiler package (version 4.0; PMID: 34557778). For pathway analysis, KEGG gene annotations were retrieved via the KEGG REST API,^6^ and KEGG enrichment analysis was similarly performed using clusterProfiler. Parameters were set to a minimum gene set size of 5 and a maximum gene set size of 5,000, with a *p*-value threshold of <0.05 and a false discovery rate (FDR) of <0.25. Data visualization was performed using the ggplot2 package.

### Venn diagram

To identify genes associated with glycolysis, the GeneCards database^7^ was queried using the keyword “Glycolysis.” Genes with a relevance score >1 were retained, yielding a total of 1,829 genes. The overlap between gene sets was visualized using the VennDiagram package in R (version 1.6.20).

### Protein–protein interaction network construction and core factor identification

Protein–protein interaction (PPI) networks of intersecting gene sets were constructed using the STRING database.^8^ Network visualization was conducted in Cytoscape software (version 3.7.2). Core functional proteins, or “hub genes,” were identified through centrality analysis, with node degree used as the primary metric to evaluate the relative importance of each node in the network.

### Analysis using Xiantao Academic platform

The Xiantao Academic platform was employed to analyze PRDX1 expression differences between tumor and normal tissues, its prognostic significance, and its correlation with 24 immune cell types using data from the TCGA-LIHC cohort.

RNA-seq data in FPKM format and corresponding clinical information were obtained from the TCGA portal^9^ and processed using the STAR alignment pipeline.

Statistical comparisons of PRDX1 expression between tumor and normal groups were performed using the Wilcoxon rank-sum test, with results visualized via the ggplot2 package in R. Survival analysis was carried out using the survival and survminer packages, incorporating proportional hazards assumption testing and Cox regression modeling.

Immune infiltration analysis was conducted using the single-sample gene set enrichment analysis (ssGSEA) algorithm implemented in the GSVA package. Immune cell signatures for 24 cell types were used to quantify infiltration scores. Correlation between PRDX1 expression and immune cell infiltration was assessed, and the results were visualized using lollipop charts generated with ggplot2.

### Cell and bacterial culture

Huh7 human hepatoma cells (HB-8065, ATCC^®^ HTB-96^™^, United States) were obtained from the American Type Culture Collection (ATCC) and maintained in high-glucose DMEM medium (11965092, Gibco, United States) supplemented with 10% fetal bovine serum (FBS) (A5670801, Gibco, United States) and 1% penicillin–streptomycin (15070063, Solarbio, China). Cells were cultured at 37°C in a 5% CO_2_ humidified incubator (Thermo Fisher Scientific, United States), and the medium was refreshed regularly to maintain optimal cell viability. When cultures reached 70–80% confluence, cells were subcultured using 0.25% trypsin-EDTA (R001100, Gibco, United States). Cell morphology and contamination were monitored using an inverted microscope (Leica Microsystems, Germany) to ensure culture integrity (Zhao et al., 2021).

*Streptococcus anginosus* (ATCC 33397) was purchased from the American Type Culture Collection and cultured on BHI agar plates (CM1136B; Thermo Fisher Scientific). For liquid culture, bacteria were grown in BHI broth (CM1135B; Thermo Fisher Scientific) at 37°C under aerobic conditions overnight.

### Cell transfection

Huh7 cells were seeded in 6-well plates at a density of 5 × 10^5^ cells per well and cultured until reaching 50–60% confluence. Transfection was performed using Lipofectamine 3000 (L3000150, Thermo Fisher Scientific, United States). Plasmid DNA and Lipofectamine 3000 were diluted in Opti-MEM medium (31985070, Gibco, United States) and incubated for 15 min to allow formation of transfection complexes, which were then added to each well. Cells were incubated at 37°C with 5% CO_2_ to facilitate transfection. Four experimental groups were established: Vector group: Huh7 cells were transfected with an empty vector (Vector, GenePharma, China); OE-PRDX1 group: Huh7 cells were transfected with a PRDX1 overexpression plasmid (OE-PRDX1, GenePharma, China); sh-NC group: Huh7 cells were transfected with a negative control plasmid (sh-NC, GenePharma, China); sh-PRDX1 group: Huh7 cells were transfected with a PRDX1 knockdown plasmid (sh-PRDX1-1 or sh-PRDX1-2, GenePharma, China). The sequences for sh-PRDX1 plasmids were as follows: sh-PRDX1-1: 5′-ATGCCAGATGGTCAGTTTAAA-3′, sh-PRDX1-2: 5′-GTGATAGGGCAGAAGAATTTA-3′.

After 48 h, cells were harvested for RT-qPCR, western blot, and other functional assays to evaluate PRDX1 expression levels and downstream effects. All transfection experiments were performed in biological triplicates to ensure data reproducibility and reliability.

### RT-qPCR

Huh7 cells were harvested 48 h post-transfection, and total RNA was extracted using TRIzol reagent (Invitrogen, United States) in accordance with the manufacturer’s protocol. Cells from each well were lysed in 1 mL of TRIzol, and RNA was isolated through isopropanol precipitation and ethanol washing. RNA purity and concentration were assessed using a NanoDrop 2000 spectrophotometer (Thermo Fisher Scientific, United States), ensuring an A260/A280 ratio between 1.8 and 2.0. cDNA synthesis was performed immediately using the PrimeScript RT reagent kit (Takara, Japan), and the resulting cDNA was stored at −20°C for subsequent analysis. Quantitative PCR was conducted using SYBR Green PCR Master Mix (Roche, Switzerland) on a LightCycler 480 system (Roche, Switzerland). Cycle threshold (Ct) values were generated using LightCycler 480 software. The ΔΔCT method was used to calculate relative mRNA expression levels of PRDX1, normalized to GAPDH as an internal control. Primer sequences were as follows:

PRDX1: (Forward: 5′-TGGGCAGCCATGAGAACAAA-3′, Reverse: 5′-GAAAGGCTGGTCTCTCCACC-3′).
GAPDH: (Forward: 5′-GTCTCCTCTGACTTCAACAGCG-3′, Reverse: 5′-ACCACCCTGTTGCTGTAGCCAA-3′).

### Western blot

Cells were washed twice with PBS (P1020, Solarbio, China) and lysed in RIPA buffer (R0020, Solarbio, China) supplemented with protease inhibitor cocktail (78442, Thermo Fisher Scientific, United States). A lysis volume of 100 μL per 1 × 10^6^ cells was used. Lysates were incubated on ice for 30 min, followed by centrifugation at 12,000 × g for 10 min at 4°C to isolate the protein-containing supernatant. Protein concentrations were determined using a BCA assay kit (P0012, Beyotime, China) and normalized to 2 mg/mL across all samples. Proteins (30 μg per sample) were separated by SDS-PAGE on 12% resolving gels and 5% stacking gels, using a PageRuler^™^ protein ladder (26617, Thermo Fisher Scientific, United States) as molecular weight reference. Electrophoresis was carried out at 100 V for stacking and 120 V for resolving. Proteins were transferred to 0.45 μm PVDF membranes (88585, Thermo Fisher Scientific, United States) using a wet transfer system at 300 mA for 90 min at 4°C. Membranes were blocked with 5% nonfat milk in TBST (T1085, Solarbio, China) for 1 h at room temperature, then incubated overnight at 4°C with the following primary antibodies (diluted in 5% milk/TBST): PRDX1 (ab257040, 1:1000, Abcam, United Kingdom), Bcl-2 (15071, 1:1000, CST, United States), Bax (2772, 1:1000, CST, United States), Caspase-3 (9662, 1:1000, CST, United States), HIF-1α (3716, 1:1000, CST, United States), LDHA (2012, 1:1000, CST, United States), PKM2 (4053, 1:1000, CST, United States), and GAPDH (2118, 1:2000, CST, United States, internal control). After primary antibody incubation, membranes were washed three times with TBST (10 min each), followed by incubation with HRP-conjugated secondary antibody (31460, 1:1000, Thermo Fisher Scientific, United States) for 1 h at room temperature. Following additional washes, protein bands were visualized using ECL substrate (P0018S, Beyotime, China) and imaged with the ChemiDoc MP system (Bio-Rad, United States). Band intensities were quantified using Image Lab software (Bio-Rad, United States) and normalized to GAPDH levels.

### CCK-8 assay

Huh7 cells were seeded in 96-well plates at a density of 5 × 10^3^ cells per well, with six replicates per group. Blank wells containing only medium and CCK-8 reagent (no cells) were included as controls. After allowing 4 h for cell attachment, the baseline optical density (OD_450_) was recorded at 0 h. At 0, 24, 48, and 72 h, 10 μL of CCK-8 reagent (96992, Sigma-Aldrich, United States) was added to each well. Plates were gently mixed and incubated at 37°C for 2 h. Absorbance at 450 nm was measured using a multifunctional microplate reader (Thermo Fisher Scientific, United States).

### Colony formation assay

To assess the clonogenic potential of Huh7 cells following transfection, 500 cells per well were seeded into 6-well plates, with three technical replicates per group. Cells were cultured in high-glucose DMEM supplemented with 10% FBS and 1% penicillin-streptomycin under standard conditions (37°C, 5% CO_2_). The medium was refreshed every 3 days to maintain optimal nutrient levels. After 10 days of incubation, cells were gently washed twice with PBS (P1020, Solarbio, China) to remove non-adherent cells and debris. Colonies were then fixed with 4% paraformaldehyde (P1110, Solarbio, China) for 15 min at room temperature, followed by two PBS washes to eliminate residual fixative. Cells were stained with 0.1% crystal violet solution (G1063, Solarbio, China) for 20 min, and excess stain was removed by rinsing under running water. The plates were air-dried at room temperature. Stained colonies, visible as purple clusters, were imaged using an inverted microscope (Olympus, Japan). Colony numbers were quantified using ImageJ software.

### Transwell migration assay

Cell migratory capacity was evaluated using a 24-well Transwell assay (8 μm pore size; 3422, Corning, United States). Inserts were pre-wetted with 200 μL serum-free high-glucose DMEM for 30 min, then the medium was discarded. Transfected Huh7 cells were suspended in serum-free medium at a density of 2 × 10^5^ cells/mL, and 200 μL of the suspension was added to the upper chamber of each insert. The lower chamber was filled with 600 μL DMEM containing 10% FBS as a chemoattractant. Chambers were incubated at 37°C in a 5% CO_2_ incubator for 24 h to allow migration. Following incubation, non-migrated cells on the upper membrane surface were gently removed with PBS. Migrated cells on the underside of the membrane were fixed with 4% paraformaldehyde for 15 min, washed twice with PBS, and stained with 0.1% crystal violet for 20 min. Excess dye was removed with running water, and membranes were air-dried. Stained cells were observed under an inverted microscope (Olympus, Japan), and five random fields were selected per well for cell counting. Images were analyzed using ImageJ software. Each experimental condition was tested in triplicate, and the experiment was independently repeated three times for reproducibility.

### Matrigel invasion assay

To assess cell invasiveness, a Matrigel-coated Transwell invasion assay was performed. Matrigel matrix (356234, BD Biosciences, United States) was thawed on ice and diluted 1:8 in serum-free DMEM. A volume of 50 μL diluted Matrigel was applied evenly to the upper surface of the Transwell insert, which was then incubated at 37°C for 1 h to allow solidification. Once solidified, 2 × 10^4^ transfected Huh7 cells in 200 μL serum-free DMEM were added to the upper chamber, and 600 μL DMEM containing 10% FBS was added to the lower chamber to serve as a chemoattractant. Cells were allowed to invade through the Matrigel for 24 h at 37°C in a 5% CO_2_. After incubation, non-invading cells were removed from the upper chamber with PBS. Invaded cells on the underside of the membrane were fixed with 4% paraformaldehyde for 15 min, washed twice with PBS, and stained with 0.1% crystal violet for 20 min. Membranes were then rinsed under running water and air-dried. Invasive cells were visualized using an inverted microscope (Olympus, Japan), and five random fields per membrane were analyzed using ImageJ software. Each group included three technical replicates, and the assay was independently repeated three times.

### Flow cytometry for apoptosis detection

Apoptosis was evaluated in Huh7 cells 48 h post-transfection. Cells were gently washed twice with PBS and resuspended to form a single-cell suspension. Apoptotic cells were stained using the Annexin V-FITC/PI apoptosis detection kit (C1062S, Beyotime, China). Stained cells were analyzed using a BD FACSCanto II flow cytometer (BD Biosciences, United States). FITC and PI fluorescence signals were excited at 488 nm, with emissions detected at 530 nm (green) and 617 nm (red), respectively. A minimum of 10,000 cells per group was acquired. Flow cytometry data were analyzed using FlowJo software (TreeStar, United States). Apoptotic stages were defined as follows: early apoptosis (Annexin V^+^/PI^−^), late apoptosis (Annexin V^+^/PI^+^), and live cells (Annexin V^−^/PI^−^).

### Lactate secretion assay

To assess lactate production, transfected Huh7 cells were seeded into 6-well plates (Corning, United States) at a density of 2 × 10^5^ cells per well and cultured for 48 h. Culture supernatants were collected into sterile centrifuge tubes and centrifuged at 13,000 × g for 10 min at 4°C to remove cell debris. The cleared supernatants were then used to measure lactate levels using a Lactate Assay Kit (S0208S, Beyotime, China). For each sample, 50 μL of supernatant was transferred into a 96-well plate, followed by the addition of 50 μL of assay reagent mixture. After mixing thoroughly, the reaction was incubated at room temperature for 30 min. A standard curve was prepared using lactate standards at final concentrations of 0, 2, 4, 6, 8, and 10 μL. Upon completion of the reaction, absorbance was measured at 570 nm using a Multiskan FC microplate reader (Thermo Fisher Scientific, United States).

### ATP content assay

ATP levels were measured in Huh7 cells 48 h post-treatment. Cells were washed twice with PBS, and ATP extraction buffer (provided with the kit) was added at a volume of 100 μL per well. Cells were lysed on ice for 5 min, and lysates were transferred to centrifuge tubes and spun at 12,000 × g for 5 min. Supernatants were collected for ATP quantification. ATP content was determined using an ATP Assay Kit (S0026, Beyotime, China). For each sample, 10 μL of supernatant was added to a 96-well plate, followed by 90 μL of detection working solution. After thorough mixing, the plate was incubated in the dark at room temperature for 5 min. Luminescence was detected using a Multiskan FC microplate reader (Thermo Fisher Scientific, United States).

### Glucose uptake assay

Huh7 cells were assigned to three experimental groups: Vector, sh-PRDX1, and sh-PRDX1 + Glu. After 48 h of culture, the culture medium was collected to measure glucose concentration, and the cells were harvested for protein extraction. Glucose levels were quantified using a glucose assay kit (361500, Rsbio, China). Glucose uptake was normalized to total protein content, which was determined using a BCA protein assay kit (23225, Thermo Fisher Scientific, United States).

### Peripheral blood mononuclear cell isolation and co-culture

Peripheral blood samples from healthy donors were diluted 1:1 with PBS and carefully layered onto a Ficoll density gradient solution (P4350, Solarbio, China). Samples were centrifuged at 2,000 rpm for 20 min at room temperature, and the peripheral blood mononuclear cell (PBMC) layer was collected. PBMCs were washed twice with PBS and resuspended in RPMI-1640 medium (11875093, Gibco, United States) supplemented with 10% FBS and IL-2 (500 U/mL; 200-02-50UG, PeproTech, United States). Cells were activated in a humidified incubator at 37°C with 5% CO_2_ for 48 h. Following activation, natural killer (NK) cell suspensions were prepared and co-cultured with Huh7 cells at an effector-to-target (E:T) ratio of 1:10 for 12 h. Huh7 cells were maintained under the same three conditions (Vector, sh-PRDX1, and sh-PRDX1 + Glu) in high-glucose DMEM medium containing 10% FBS and 1% penicillin-streptomycin.

### Lactate dehydrogenase release assay

Lactate dehydrogenase (LDH) activity in the culture supernatant was assessed using a LDH release assay kit (C0016, Beyotime, China). LDH release was expressed as a percentage of total LDH activity, calculated by comparing supernatant levels with total LDH measured in lysed cell controls.

### NK cell activity assay

Following co-culture, NK cells were collected and adjusted to an appropriate concentration. Cells were stained with anti-CD107a-FITC antibody (328605, BioLegend, United States) and anti-IFN-γ-PE antibody (502508, BioLegend, United States) and incubated at 37°C in the dark for 30 min. After staining, cells were fixed in 75% ethanol, washed twice with PBS, and resuspended as a single-cell suspension. Fluorescence signals were measured using a BD FACSCanto II flow cytometer (BD Biosciences, United States). Expression levels of CD107a and IFN-γ were quantified to assess NK cell activation.

### ELISA for inflammatory cytokines

After 12 h of co-culture, supernatants were collected and centrifuged at 1,200 × g for 5 min to remove cellular debris. Samples were stored at −80°C until analysis. Concentrations of IL-6 and TNF-α were measured using ELISA kits (IL-6: SEKH-0013; TNF-α: SEKH-0047; Solarbio, China), following the manufacturer’s protocols. Briefly, 100 μL of standards and diluted samples were added to ELISA plate wells and incubated at 37°C for 1 h. After three washes, biotin-labeled antibody working solution was added and incubated at room temperature for 30 min. After another wash, enzyme-labeled conjugate was added and incubated for 15 min at room temperature. Substrate solution was then added and incubated in the dark for 15 min, followed by addition of stop solution. Absorbance at 450 nm (OD450) was measured using a Multiskan FC microplate reader (Thermo Fisher Scientific, United States).

### NKG2D ligand detection

To assess the surface expression of NKG2D ligands, Huh7 cells were harvested and stained with the following antibodies: anti-MICA/B-FITC (MA5-38727, Thermo Fisher Scientific, United States) and ULBP1/2-PE (ULBP1: FAB1380P; ULBP2: FAB1298P, Bio-Techne, United States). Cells were incubated with antibodies for 30 min at room temperature, followed by two washes with PBS. A single-cell suspension was prepared, and fluorescence intensity was measured using a flow cytometer. The expression levels of NKG2D ligands were quantified accordingly.

### Immunofluorescence assay

Cells were washed twice with PBS and fixed with 4% paraformaldehyde (P1110, Solarbio, China) for 15 min at room temperature. After fixation, cells were washed three times with PBS and permeabilized with 0.3% Triton X-100 (T8200, Solarbio, China) for 10 min. Non-specific binding was blocked using 5% bovine serum albumin (BSA) (A8010, Solarbio, China) for 1 h at room temperature. Cells were then incubated overnight at 4°C with the primary antibody against PRDX1 (ab266842, 1:200, Abcam, United Kingdom). The following day, after three PBS washes, cells were incubated with a FITC-conjugated secondary antibody (ab6785, 1:500, Abcam, United Kingdom) for 1 h at room temperature in the dark. Nuclei were stained with DAPI (ab104139, 1:200, Abcam, United Kingdom) for 5 min in the dark at room temperature. After three additional PBS washes, coverslips were mounted using antifade mounting medium (S2110, Solarbio, China). Fluorescence images were captured using a Leica SP8 confocal microscope (Leica Microsystems, Germany) with appropriate excitation/emission settings. Five random fields per sample were selected for imaging, and fluorescence intensity was quantified using ImageJ software (NIH, United States).

### Establishment of a subcutaneous tumor-bearing mouse model

Female BALB/c-nu/nu nude mice (4 weeks old, 18–20 g) were purchased from Beijing HFK Bioscience Co., Ltd. Mice were housed in a sterile environment with a controlled temperature (22–24°C), humidity (50–60%), and a 12-h light/dark cycle, with free access to sterile food and water. Huh7 cells in the logarithmic growth phase were resuspended in PBS (pH 7.4) at a concentration of 2 × 10^6^ cells/100 μL. A total of 100 μL of cell suspension was subcutaneously injected into the right dorsal flank of each mouse using a sterile syringe. Mice were randomly divided into five groups (*n* = 8 per group): Control group: Tumor-bearing mice without Bacilli (*Streptococcus anginosus*) infection; Bacilli group: Tumor-bearing mice infected with Bacilli (*Streptococcus anginosus*) at a multiplicity of infection (MOI) of 10; Bacilli + sh-PRDX1 group: Tumor-bearing mice infected with Bacilli and transduced with PRDX1 shRNA; Bacilli + 2-deoxy-D-glucose (2-DG) group: Tumor-bearing mice infected with Bacilli and treated with the glycolysis inhibitor 2-DG (10 mg/kg, D8375, Sigma-Aldrich, United States); Bacilli + PD-1 group: Tumor-bearing mice infected with Bacilli and treated with the PD-1 inhibitor (10 mg/kg, HY-134886, MCE, United States). Tumor length and width were measured every 3 days using a Vernier caliper, and tumor volume was calculated using the formula: Tumor volume (mm^3^) = 0.5 × length (mm) × width^2^ (mm). Tumor growth was monitored over a 28-day period (Nakatake et al., 2018).

### Data analysis

Data were analyzed using SPSS software (version 27, IBM, United States). Independent sample *t*-tests were conducted to compare EPDS and NRS scores between the two groups. Chi-square tests were used to analyze differences in adverse event incidence between groups. Statistical significance was set at *p* < 0.05.

## Results

### Changes in gut microbiota characteristics and their potential biological significance in HCC patients

Emerging evidence indicates that the gut microbiome plays a pivotal role in the progression of liver diseases, including the development of HCC (Yu and Schwabe, 2017). To explore the molecular mechanisms by which gut microbiota contribute to HCC pathogenesis, we first compared the gut microbiota composition between patients with HCC and healthy individuals.

We retrieved the 16S rRNA sequencing dataset PRJNA1127013 from the SRA database, which comprises 17 HCC samples (all classified as grade III or higher) and 12 healthy control samples. Rarefaction curve analysis indicated that microbial richness plateaued as sampling depth increased in both groups (Supplementary Figure S1A), suggesting sufficient sequencing coverage to capture the majority of microbial species present.

**Figure 1:**
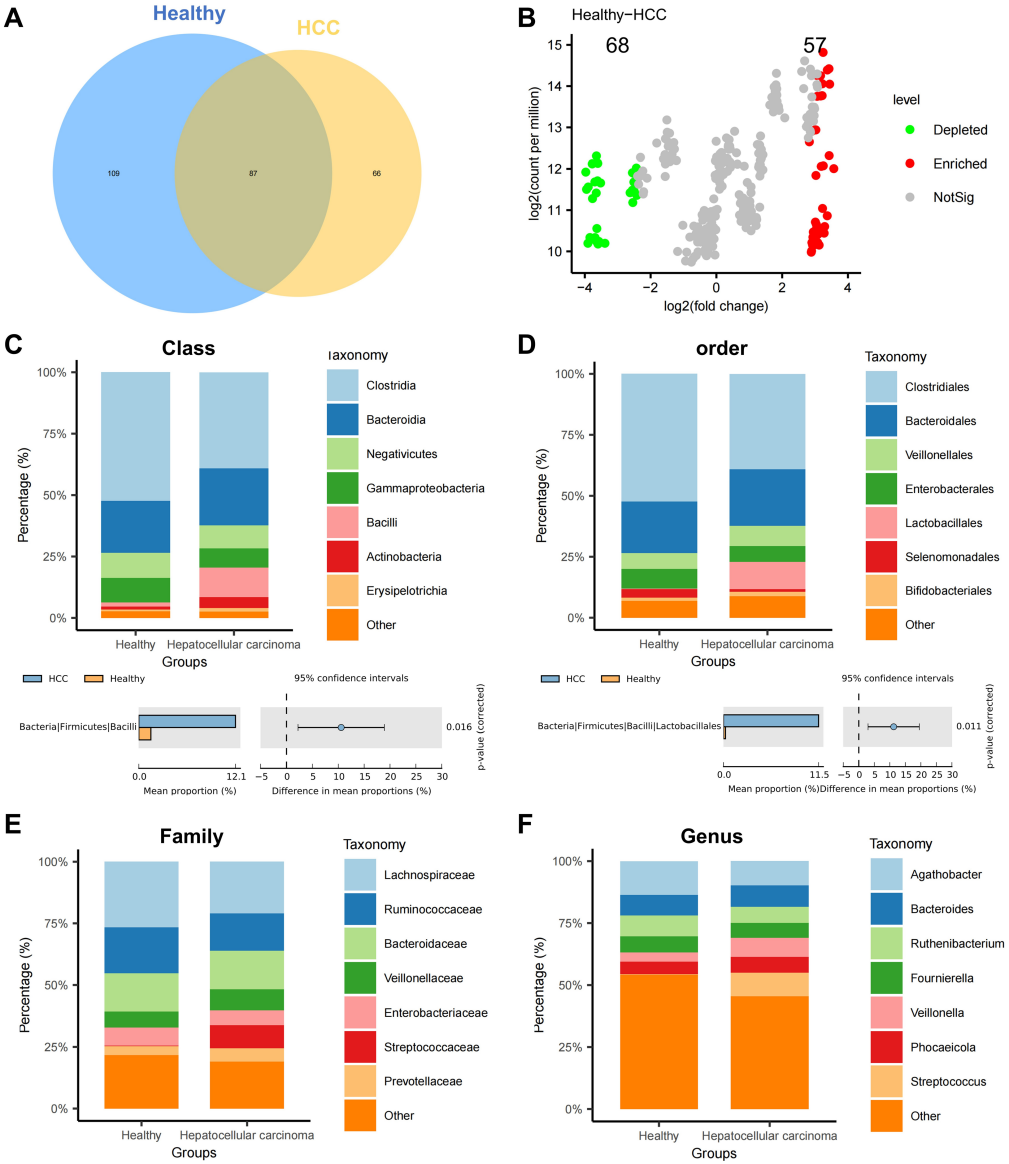
Differential analysis of gut microbiota composition at various taxonomic levels in the HCC and Healthy groups. **(A)** Venn diagram showing the intersection of OTUs between the HCC and Healthy groups. **(B)** Volcano plot comparing abundance differences between the HCC and Healthy groups. **(C–F)** Stacked bar charts of relative abundance at the class, order, family, and genus levels, with groups on the x-axis. Different colors represent gut microbiota at different phyla levels. The lower panels show species abundance difference analysis at the class, order, family, and genus levels; Healthy: *n* = 12; HCC: *n* = 17.

Alpha diversity metrics—including Chao1, Inverse Simpson, Richness, Shannon, Simpson, and ACE indices—were employed to assess within-sample diversity. No statistically significant differences in species richness were observed between the HCC and healthy groups (Supplementary Figures S1B–G). However, Venn diagram analysis of operational taxonomic units (OTUs) revealed distinct microbial compositions, with 66 OTUs unique to the HCC group and 109 OTUs unique to the Healthy group (Figure 1A).

To examine intergroup variation, we performed PCoA based on beta diversity, which revealed a moderate degree of spatial separation between HCC and healthy samples, particularly along principal components 1 and 2 (Supplementary Figure S1H). Differential abundance analysis was conducted using the edgeR package in R. At the phylum level, Manhattan plots illustrated significant OTU-level differences between groups (Supplementary Figure S1I). A volcano plot further identified 68 significantly downregulated and 57 significantly upregulated OTUs in the HCC group relative to controls (Figure 1B).

We next assessed microbial composition at multiple taxonomic levels—phylum, class, order, family, and genus—using stacked bar plots. At the phylum level, both groups were predominantly composed of Firmicutes, Bacteroidetes, and Proteobacteria, with no significant group-wise differences (Supplementary Figure S1J). At the class level, Clostridia, Bacteroidia, and Negativicutes dominated; however, the relative abundance of Bacilli was significantly elevated in the HCC group (Figure 1C). At the order level, most taxa were assigned to Clostridiales, Bacteroidales, and Veillonellales, with a marked increase in Lactobacillales in HCC (Figure 1D). At the family level, Lachnospiraceae, Ruminococcaceae, and Bacteroidaceae were most abundant overall, while Streptococcaceae was significantly enriched in the HCC group (Figure 1E). At the genus level, Streptococcus was significantly upregulated in HCC, whereas Anaerotaenia, Anaerobutyricum, and Merdimonas were more prevalent in healthy individuals (Figure 1F).

Collectively, these findings demonstrate substantial alterations in gut microbial diversity and composition in HCC patients, implicating microbial dysbiosis as a potential contributor to HCC progression.

To contextualize these findings within the broader scope of our research, we created an integrated schematic diagram outlining the full analytical workflow—from public database mining and 16S rRNA sequencing to single-cell transcriptomics and functional validation (Supplementary Figure S2). This overview provides a conceptual bridge between microbiota profiling and subsequent mechanistic investigations.

**Figure 2:**
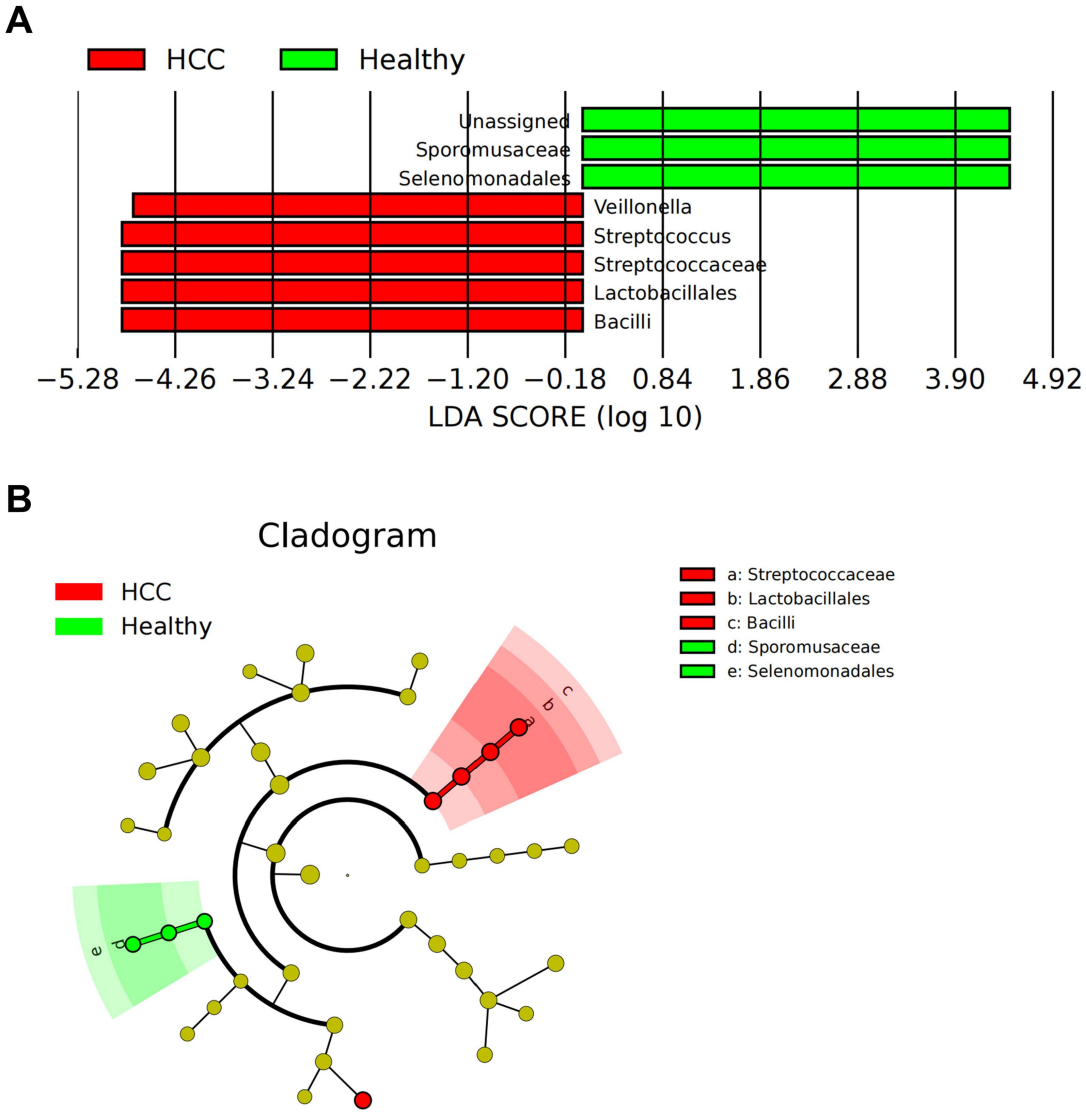
LEfSe-based analysis of gut microbiota composition and functional characteristics in HCC patients. **(A)** LDA value distribution bar chart of species abundance in the HCC and Healthy groups. **(B)** Phylogenetic tree of species abundance in the HCC and Healthy groups. Circles radiating from the center represent taxonomic levels from phylum to genus. The circle diameter corresponds to relative abundance, with yellow nodes indicating species with no significant differences, red nodes representing microbial groups with higher abundance in the HCC group, and green nodes representing those with higher abundance in the Healthy group; Healthy: *n* = 12; HCC: *n* = 17.

We further employed LEfSe analysis at the genus level to pinpoint discriminatory taxa. Using an LDA score threshold of log10 > 2, we found that Bacilli, Lactobacillales, and Streptococcaceae were significantly enriched in HCC fecal samples, whereas Selenomonadales and Sporomusaceae were more abundant in healthy controls (Figures 2A,B).

In summary, our comprehensive multi-level analysis revealed distinct gut microbial signatures in HCC patients, especially notable increases in Bacilli, Lactobacillales, and Streptococcaceae.

### Gut microbiota-associated metabolic pathways in HCC patients are enriched in pyruvate metabolism and glycolysis

**Figure 3:**
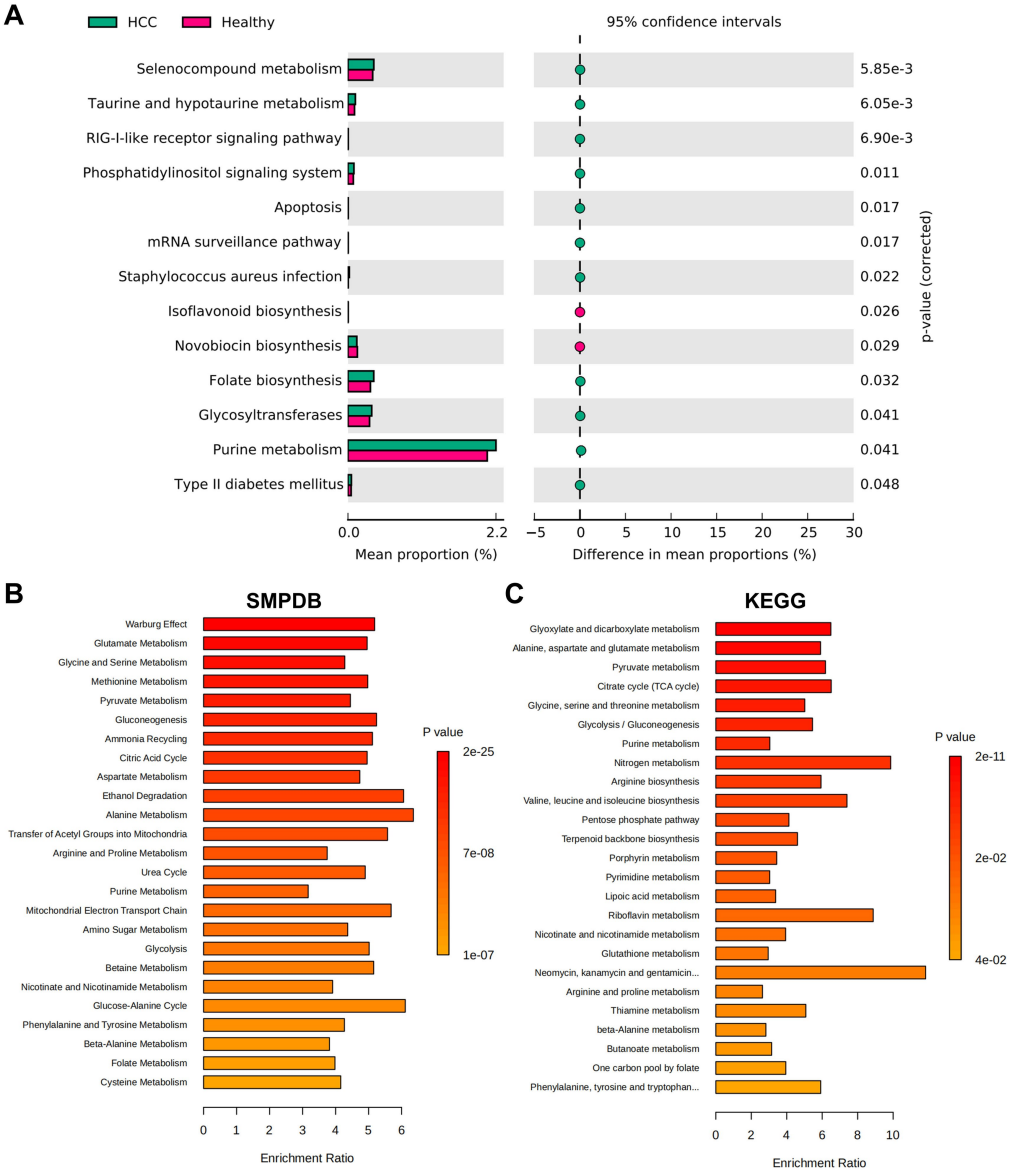
Functional pathway analysis of HCC based on microbiota metabolism characteristics. **(A)** KEGG functional enrichment prediction performed using PICRUSt. **(B,C)** Functional enrichment analysis results from the MetaboAnalyst database. **(B)** Shows the SMPDB pathways. **(C)** Shows the KEGG pathways.

Following the identification of distinct gut microbiota compositions between HCC patients and healthy individuals, we further explored the functional implications of these microbial differences by assessing associated metabolic pathways and biochemical activities. Functional predictions were performed using PICRUSt, followed by KEGG pathway enrichment analysis. In the HCC group, several pathways were significantly upregulated, including Selenocompound metabolism, Taurine and hypotaurine metabolism, and the RIG-I-like receptor signaling pathway. In contrast, pathways such as Isoflavonoid biosynthesis and Novobiocin biosynthesis were markedly downregulated (Figure 3A). To investigate the metabolic relevance of key bacterial taxa, we queried the gutMGene database^10^ for metabolites associated with Bacilli, Lactobacillales, and Streptococcaceae. A total of 320 metabolites were associated with Bacilli and 332 metabolites with Streptococcaceae. No metabolite data were available for Lactobacillales. After removing overlaps, we obtained 339 unique metabolites collectively linked to Bacilli and Streptococcaceae.

To assess the functional relevance of these metabolites, enrichment analysis was conducted using the MetaboAnalyst platform.^11^ SMPDB-based analysis revealed that the identified metabolites were primarily enriched in pathways such as the Warburg effect, glutamate metabolism, glycine and serine metabolism, methionine metabolism, and pyruvate metabolism (Figure 3B). Similarly, KEGG pathway analysis indicated significant enrichment in glyoxylate and dicarboxylate metabolism, alanine, aspartate, and glutamate metabolism, pyruvate metabolism, the citrate cycle (TCA cycle), and glycolysis/gluconeogenesis (Figure 3C).

By integrating microbiota-derived functional predictions with metabolic pathway analysis, our study revealed that HCC-associated microbial taxa were closely linked to alterations in host metabolic pathways. In particular, enrichment in glycolytic and pyruvate-related pathways, as well as the Warburg effect, underscores the potential role of microbial metabolites in reprogramming tumor energy metabolism. These altered metabolic routes may provide both bioenergetic support and molecular cues to promote HCC development and immune modulation. Overall, these findings suggest a mechanistic link between microbial dysbiosis and host metabolic reprogramming in HCC.

### Reduced proportion of NK cells and reconstruction of the immunosuppressive network in the HCC microenvironment

Building upon the observed alterations in gut microbiota in HCC patients, we next investigated the cellular architecture and intercellular interactions within the HCC microenvironment using single-cell transcriptomic data. Data were obtained from the GEO database (GSE189903), including tumor core tissue samples from four HCC patients (each with >8,000 cells) and adjacent non-tumor tissue from three individuals (also with >8,000 cells). Data preprocessing and integration were conducted using the Seurat package in R. High-quality cells were filtered using the following criteria: nFeature_RNA < 5,000, nCount_RNA < 20,000, and percent.mt < 20%. This yielded a final expression matrix consisting of 18,228 genes across 98,343 cells (Supplementary Figure S3A). Correlation analysis revealed a strong positive correlation (*r* = 0.91) between nCount_RNA and nFeature_RNA, and a negligible correlation (*r* = −0.02) between nCount_RNA and percent.mt (Supplementary Figure S3B), indicating high-quality single-cell data suitable for downstream analysis.

Following data normalization and selection of highly variable genes, we performed PCA for linear dimensionality reduction (Supplementary Figure S3C). Visualization along PC_1 and PC_2 revealed the presence of batch effects (Supplementary Figure S3D).

To address this, the Harmony algorithm was applied for batch correction, and ElbowPlot was used to rank principal components by standard deviation (Supplementary Figures S3E,F). Post-correction, batch effects were largely eliminated (Supplementary Figure S3G), validating the integration process. Subsequently, nonlinear dimensionality reduction using the UMAP algorithm was applied to the top 20 PCs. Cell clustering at multiple resolutions was visualized using the clustree package, and the final UMAP projection delineated 34 distinct cell clusters (Supplementary Figures S4A–C).

**Figure 4:**
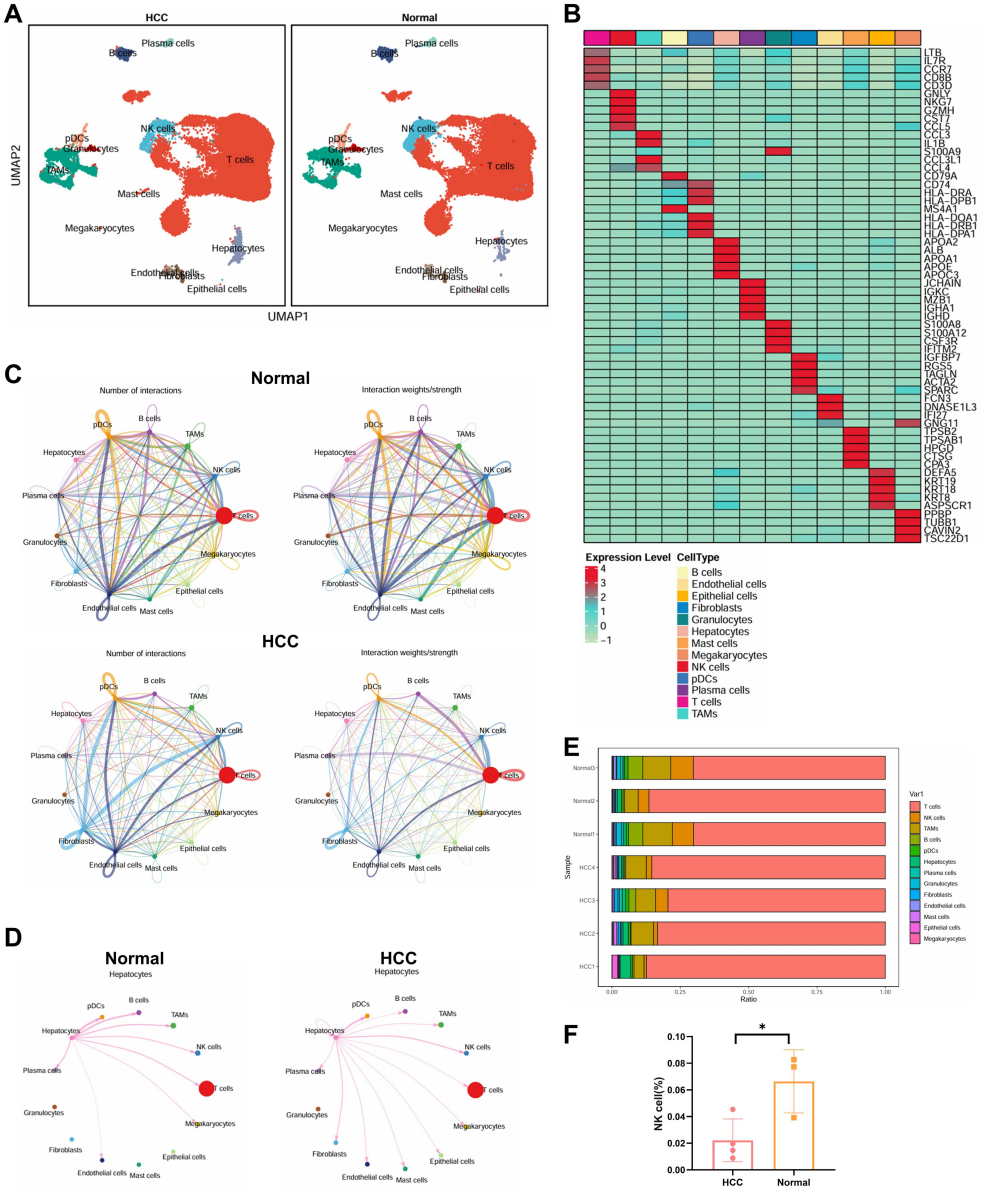
Single-cell transcriptomic analysis of immune microenvironmental cell communication and functional changes in HCC patients. **(A)** Visualization of cell annotations based on UMAP clustering results. **(B)** Correlation heatmap of the top five genes expressed in 13 cell types. **(C)** Cell communication circle plot, with the left panel representing pathway quantity (line thickness), and the right panel representing interaction strength (line thickness). **(D)** Detailed interactions between hepatocytes and other cell types. **(E)** Proportional distribution of 13 cell types in each sample. **(F)** Differential analysis of NK cell abundance between the two groups, with * indicating *p* < 0.05. Normal: *n* = 3; HCC: *n* = 4.

These clusters were annotated using the SingleR package in conjunction with manual curation, resulting in the identification of 13 distinct cell types (Figure 4A). A heatmap displaying the top five marker genes for each cell type supported the accuracy of the clustering (Figure 4B).

To evaluate intercellular communication, we used the CellChat package. Compared to adjacent normal tissue, the HCC group exhibited markedly reduced immune cell interactions, indicative of a globally immunosuppressive microenvironment (Figure 4C). Notably, hepatocytes in HCC displayed enhanced interactions with NK cells and B cells, but reduced interactions with tumor-associated macrophages (TAMs) and T cells (Figure 4D). Quantitative analysis of immune cell proportions revealed a statistically significant reduction in NK cell populations within the HCC samples (Figures 4E,F).

Overall, our single-cell transcriptomic analysis provides a high-resolution map of the cellular and functional immune landscape in HCC. The reduction in NK cell abundance, combined with altered intercellular signaling patterns, highlights the reconstruction of an immunosuppressive network that may contribute to immune evasion and tumor progression in HCC.

### PRDX1 promotes immune evasion via glycolytic pathways and reduces NK cell infiltration

**Figure 5:**
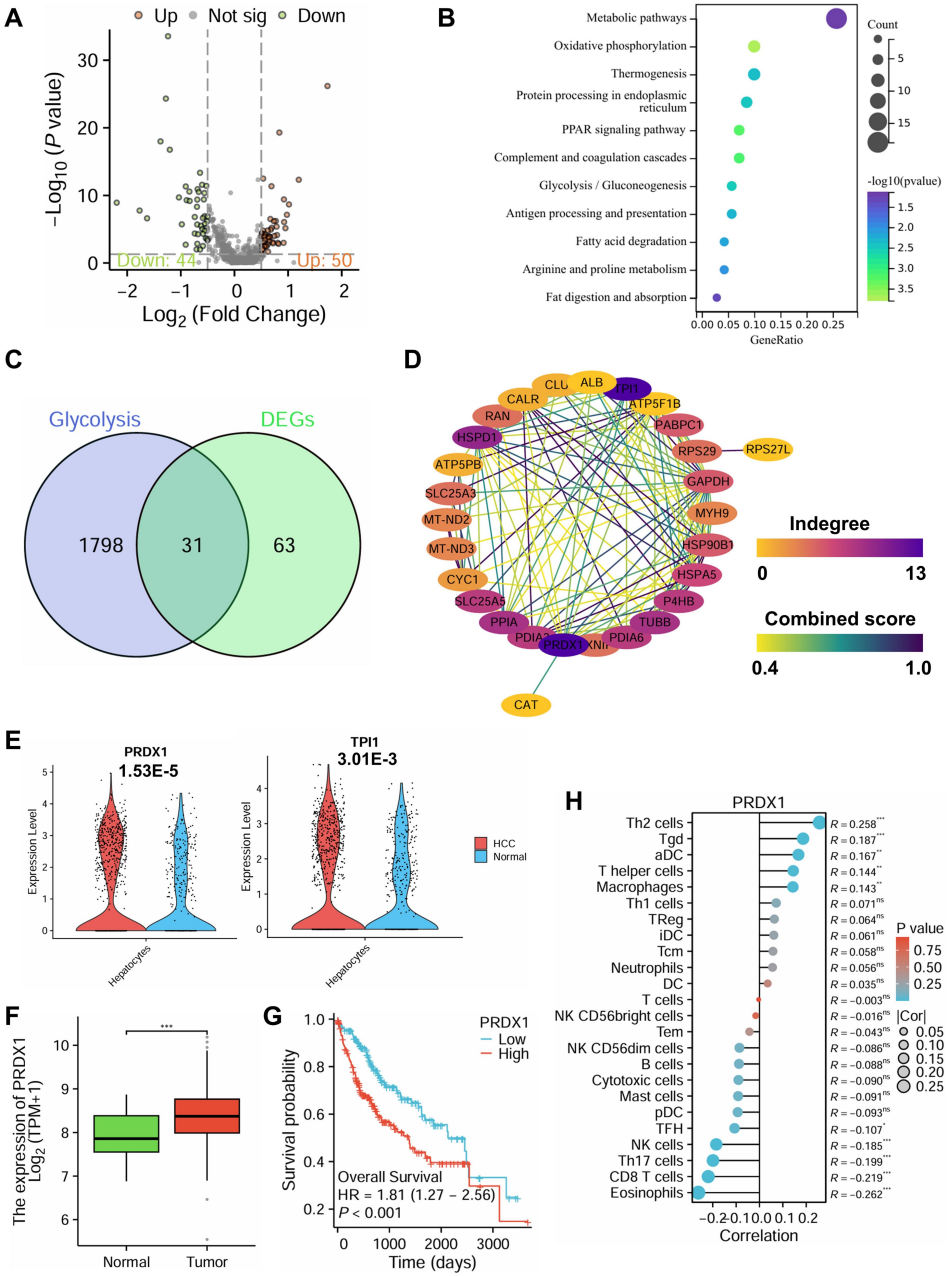
Role of PRDX1-mediated glycolytic pathways in immune evasion in HCC based on single-cell multiomics. **(A)** Volcano plot of differential analysis in hepatocytes from the scRNA-seq dataset, with red indicating significantly upregulated genes, green indicating significantly downregulated genes, and gray indicating genes with no significant change. **(B)** KEGG functional enrichment analysis of DEGs. **(C)** Venn diagram of glycolysis-related genes and DEGs. **(D)** PPI analysis of the 31 intersecting genes. **(E)** Differential expression analysis of PRDX1 and TPI1 in HCC and normal tissues from the scRNA-seq dataset. **(F)** Differential expression analysis of PRDX1 in the TCGA-LIHC dataset, *** indicating *p* < 0.001. **(G)** Survival analysis of PRDX1 in the TCGA-LIHC dataset. **(H)** Correlation analysis of PRDX1 with 24 immune cell types in the TCGA-LIHC dataset, represented by a lollipop plot.

To further elucidate the molecular mechanisms underlying immune evasion in HCC, we performed differential expression analysis on hepatocyte clusters extracted from scRNA-seq data. A total of 94 DEGs were identified, including 50 upregulated and 44 downregulated genes in hepatocytes from HCC tissues compared to non-tumor controls (Figure 5A).

KEGG pathway enrichment analysis revealed that these DEGs were significantly enriched in metabolic pathways, oxidative phosphorylation, PPAR signaling, and glycolysis/gluconeogenesis (Figure 5B).

In GO enrichment, biological process (BP) terms included establishment of localization, immune response, and leukocyte-mediated immunity (Supplementary Figure S5A); cellular component (CC) terms were enriched in the extracellular region, extracellular space, and extracellular vesicles (Supplementary Figure S5B); and molecular function (MF) terms included oxidoreductase activity, enzyme inhibitor activity, and lipoprotein particle receptor binding (Supplementary Figure S5C).

Integration of 16S rRNA microbiome sequencing and scRNA-seq transcriptomic data consistently highlighted glycolysis as a key pathway enriched in HCC. Functional predictions using PICRUSt indicated that microbial communities in HCC patients possess elevated glycolytic potential. Moreover, MetaboAnalyst-based enrichment analysis of 339 metabolites associated with Bacilli, Lactobacillales, and Streptococcaceae also demonstrated enrichment in glycolysis, pyruvate metabolism, and the TCA cycle. These findings were consistent with KEGG analysis of DEGs from tumor hepatocytes, which showed upregulation of glycolytic signaling pathways.

Glycolysis is a hallmark of tumor metabolism (Chelakkot et al., 2023), fueling rapid proliferation by supplying ATP and biosynthetic precursors. Importantly, glycolytic byproducts such as lactate contribute to immune evasion by suppressing NK cell cytotoxicity, in part by downregulating NKG2D receptor expression (Guo et al., 2022; Brand et al., 2016). Collectively, these findings suggest that gut microbiota-mediated metabolic reprogramming may enhance glycolysis in hepatocytes, thereby reshaping the TIME to favor immune evasion.

To explore key glycolysis-related regulators, we retrieved glycolysis-associated genes from the GeneCards database and intersected them with our DEGs, yielding 31 overlapping genes (Figure 5C). PPI network analysis using the STRING database identified PRDX1 and TPI1 as central hub genes with the highest degree of interaction (Figure 5D). Further analysis of scRNA-seq data showed that both genes were significantly upregulated in hepatocytes from the HCC group, with PRDX1 exhibiting the most pronounced differential expression (Figure 5E).

Validation using the Xiantao Academic platform based on TCGA-LIHC RNA-seq data confirmed that PRDX1 expression was markedly higher in tumor tissues compared to adjacent normal liver tissues (Figure 5F). Moreover, patients with high PRDX1 expression had significantly worse overall survival (Figure 5G). Immune infiltration analysis revealed that PRDX1 expression negatively correlated with NK cell infiltration, among other immune cell types (Figure 5H).

Taken together, this multi-omics integration highlights the pivotal role of PRDX1-mediated glycolytic activity in promoting immune evasion in HCC. Elevated PRDX1 expression is associated with poor prognosis and reduced NK cell infiltration, indicating that PRDX1 may serve as a key regulator of tumor immune evasion and represents a promising therapeutic target in HCC.

### Validation of PRDX1’s role in modulating HCC cell phenotypes *in vitro*

**Figure 6:**
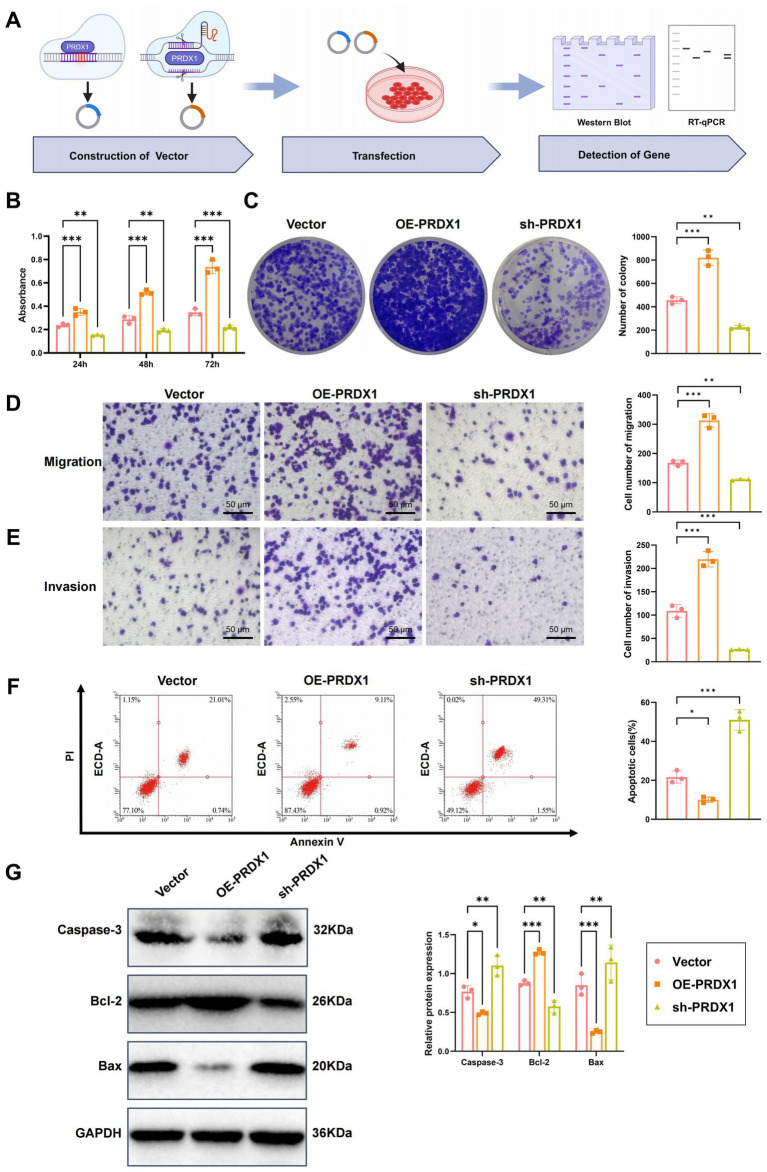
Effects of PRDX1 on proliferation, migration, invasion, and apoptosis in Huh7 cells. **(A)** Diagram illustrating the experimental workflow. **(B)** CCK-8 assay to assess the effect of OE-PRDX1 and sh-PRDX1 on Huh7 cell proliferation. **(C)** Clonogenic assay to evaluate the effect of OE-PRDX1 and sh-PRDX1 on Huh7 cell clonogenicity. **(D)** Transwell assay to assess the effect of OE-PRDX1 and sh-PRDX1 on Huh7 cell migration (scale bar = 50 μm). **(E)** Matrigel-coated Transwell assay to evaluate the effect of OE-PRDX1 and sh-PRDX1 on Huh7 cell invasion (scale bar = 50 μm). **(F)** Annexin V-FITC/PI double-staining flow cytometry to examine the impact of OE-PRDX1 and sh-PRDX1 on Huh7 cell apoptosis rates. **(G)** Western blot analysis to determine the effect of OE-PRDX1 and sh-PRDX1 on the expression of anti-apoptotic protein Bcl-2 and pro-apoptotic proteins Bax and activated Caspase-3. All cell experiments were performed in triplicate, with * indicating *p* < 0.05, ** indicating *p* < 0.01, and *** indicating *p* < 0.001.

To elucidate the functional role of PRDX1 in HCC progression, we assessed its effects on cell proliferation, migration, invasion, and apoptosis in Huh7 hepatocellular carcinoma cells. PRDX1 was either overexpressed or silenced in Huh7 cells, as outlined in the experimental workflow (Figure 6A). To verify the efficiency of genetic modulation, RT-qPCR and western blotting were performed. As expected, PRDX1 mRNA and protein levels were markedly increased in the PRDX1 overexpression group (OE-PRDX1) compared to the Vector control (Supplementary Figure S6A). Among the two shRNA constructs tested, sh-PRDX1-1 exhibited superior silencing efficiency compared to sh-PRDX1-2, and was therefore selected for subsequent experiments (Supplementary Figure S6B).

Cell proliferation was measured using the CCK-8 assay. OE-PRDX1 cells demonstrated significantly enhanced proliferation compared to the Vector group, with OD450 values increasing by approximately 1.5-, 1.8-, and 2.2-fold at 24, 48, and 72 h, respectively. In contrast, PRDX1 knockdown (sh-PRDX1) significantly suppressed cell proliferation (Figure 6B).

Consistent with these findings, the colony formation assay showed a notable increase in colony numbers in the OE-PRDX1 group (92 ± 7 colonies), while the sh-PRDX1 group exhibited significantly fewer colonies (36 ± 5 colonies) (Figure 6C), supporting PRDX1’s role in promoting HCC cell proliferation.

The effects of PRDX1 on cell migration and invasion were evaluated using Transwell assays. In the migration assay, the number of migrating cells was significantly higher in the OE-PRDX1 group, whereas sh-PRDX1 reduced migration (Figure 6D). In the Matrigel invasion assay, PRDX1 overexpression markedly enhanced invasion, while knockdown of PRDX1 resulted in an invasion rate that was only 31% of that observed in control cells (Figure 6E), suggesting a strong role of PRDX1 in facilitating tumor cell invasiveness.

To evaluate the impact of PRDX1 on apoptosis, we employed Annexin V-FITC/PI dual staining followed by flow cytometry in an H_2_O_2_-induced apoptosis model. The apoptosis rate was significantly lower in OE-PRDX1 cells than in the control group, indicating a protective anti-apoptotic effect (Figure 6F).

Western blot analysis further supported these findings: the anti-apoptotic protein Bcl-2 was significantly upregulated in the OE-PRDX1 group, whereas pro-apoptotic proteins Bax and cleaved Caspase-3 were markedly downregulated. Conversely, sh-PRDX1 reduced Bcl-2 expression and increased Bax and cleaved Caspase-3 levels (Figure 6G).

These results demonstrate that PRDX1 significantly enhances the proliferation, migration, and invasion of HCC cells while suppressing apoptosis.

### *In vitro* validation of PRDX1-mediated regulation of glycolytic pathways and its impact on NK cell cytotoxicity

**Figure 7:**
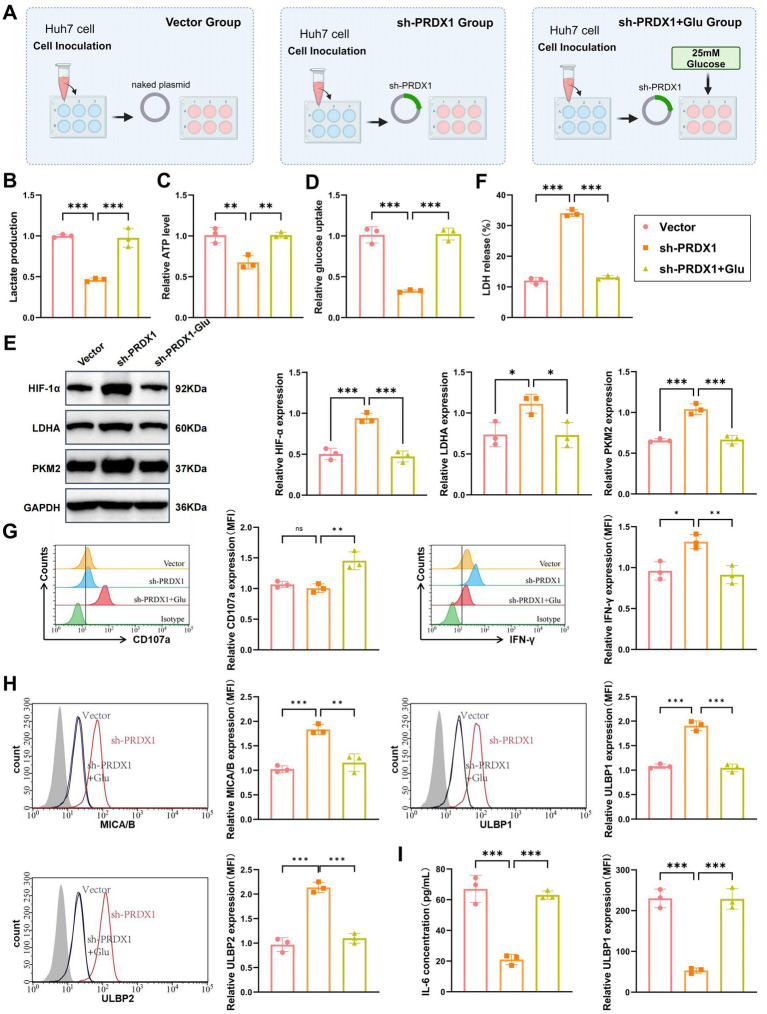
*In vitro* validation of PRDX1-mediated regulation of the glycolysis pathway and its effect on NK cell cytotoxicity. **(A)** Diagram illustrating the experimental workflow. **(B)** Lactic acid levels in the culture supernatant of sh-PRDX1 and sh-PRDX1 + Glu Huh7 cells, measured using a lactic acid assay kit (enzymatic method). **(C)** ATP content in sh-PRDX1 and sh-PRDX1 + Glu Huh7 cells, measured using an ATP assay kit. **(D)** Glucose uptake in sh-PRDX1 and sh-PRDX1 + Glu Huh7 cells, measured using a glucose assay kit. **(E)** Western blot analysis of HIF-1α, LDHA, and PKM2 protein expression in sh-PRDX1 and sh-PRDX1 + Glu Huh7 cells. **(F)** LDH release assay to assess the effect of sh-PRDX1 and sh-PRDX1 + Glu on NK cell-mediated lysis of Huh7 cells. **(G)** Flow cytometry analysis to evaluate the expression of NK cell activation markers CD107a and IFN-γ in sh-PRDX1 and sh-PRDX1 + Glu NK cells. **(H)** Flow cytometry analysis of NKG2D ligands (MICA/B and ULBP1/2) expression in Huh7 cells after sh-PRDX1 and sh-PRDX1 + Glu. **(I)** ELISA to measure the levels of inflammatory cytokines (IL-6 and TNF-α) in the supernatant of co-culture systems with sh-PRDX1 and sh-PRDX1 + Glu. All cell experiments were performed in triplicate, with * indicating *p* < 0.05, ** indicating *p* < 0.01, and *** indicating *p* < 0.001.

To determine whether silencing PRDX1 enhances NK cell cytotoxicity and reverses immune evasion by inhibiting glycolysis in HCC cells, we conducted a series of *in vitro* experiments. The following three experimental groups were established: Vector group (Huh7 cells transfected with an empty vector), sh-PRDX1 group (Huh7 cells transfected with sh-PRDX1), and sh-PRDX1 + Glu group [Huh7 cells transfected with sh-PRDX1 with exogenous addition of high-concentration glucose (25 mM) to restore glycolysis]. A schematic of the experimental design is presented in Figure 7A.

Using an enzymatic lactate assay, we found that lactate secretion was significantly reduced in the sh-PRDX1 group to approximately 40–50% of the Vector group. Lactate levels were partially restored in the sh-PRDX1 + Glu group (Figure 7B).

Intracellular ATP levels, quantified via an ATP assay kit, showed a significant reduction in the sh-PRDX1 group, reaching approximately 30–50% of control levels. ATP production was partially restored in the sh-PRDX1 + Glu group (Figure 7C).

Glucose uptake was also significantly suppressed in the sh-PRDX1 group and partially recovered in the glucose-rescue group, as measured by a glucose assay (Figure 7D). Western blot analysis further confirmed the glycolytic suppression, with marked downregulation of HIF-1*α*, LDHA, and PKM2 proteins in the sh-PRDX1 group, while their expression partially returned to baseline levels in the sh-PRDX1 + Glu group (Figure 7E).

NK cells were isolated from healthy donor PBMCs using Ficoll gradient centrifugation, followed by IL-2 (500 U/mL) stimulation for 48 h. Activated NK cells were co-cultured with Huh7 cells at an effector-to-target (E:T) ratio of 1:10 for 12 h. LDH release assays demonstrated a significantly higher NK cell-mediated lysis rate in the sh-PRDX1 group, which was partially attenuated in the glucose-rescued condition (Figure 7F).

To further assess NK cell activation, flow cytometry was performed to detect CD107a and IFN-*γ* expression. Both markers were significantly upregulated in the sh-PRDX1 group, indicating enhanced cytotoxic function, and partially restored to baseline levels in the sh-PRDX1 + Glu group (Figure 7G). Additionally, NKG2D ligand expression was analyzed by flow cytometry. MICA/B and ULBP1/2 surface expression levels were significantly elevated in the sh-PRDX1 group and partially decreased following glucose supplementation (Figure 7H).

Finally, ELISA quantification of inflammatory cytokines in the co-culture supernatant revealed significantly reduced IL-6 and TNF-α levels in the sh-PRDX1 group, with partial recovery observed in the sh-PRDX1 + Glu group (Figure 7I).

These findings collectively demonstrate that PRDX1 knockdown suppresses glycolytic metabolism, enhances NK cell cytotoxicity, upregulates NKG2D ligands, and reduces inflammatory cytokine production. This provides mechanistic insight into the link between tumor glycolysis and immune evasion in HCC.

### Bacilli infection promotes immune evasion in HCC by mediating glycolytic pathways through upregulation of PRDX1

Bioinformatic analyses revealed a significant enrichment of *Bacilli* and *Streptococcaceae* in HCC patients (Figures 2A,B). These bacterial taxa may modulate glycolytic pathways and the TIME through their metabolic byproducts, with PRDX1 and glycolysis acting as key functional nodes. To further elucidate the interactions among specific bacterial infections, PRDX1 expression, glycolysis, and immunotherapeutic response, we conducted both *in vitro* and *in vivo* studies using Huh7 cells.

Huh7 cells were infected with *Bacilli* or *Streptococcaceae* following the experimental workflow shown in Supplementary Figure S7A. RT-qPCR analysis demonstrated a significant upregulation of PRDX1 mRNA levels following *Bacilli* infection, whereas *Streptococcaceae* infection induced no significant changes (Supplementary Figure S7B). Western blotting confirmed these findings at the protein level, with *Bacilli* infection markedly increasing PRDX1 protein expression, in contrast to the *Streptococcaceae*-infected and control groups, which showed no substantial difference (Supplementary Figure S7C).

Flow cytometry analysis revealed that *Bacilli* infection led to a 62% reduction in mean fluorescence intensity (MFI) of the NKG2D ligand MICA/B and a 47% decrease in ULBP1/2 expression on the surface of Huh7 cells. Conversely, *Streptococcaceae* infection only modestly reduced MICA/B and ULBP1/2 expression by 21 and 15%, respectively (Supplementary Figure S7D).

Immunofluorescence staining further indicated that in the *Bacilli* infection group, PRDX1 was predominantly localized in the cytoplasm, with fluorescence intensity increasing by 4.6-fold compared to the control (*p* < 0.001). In contrast, *Streptococcaceae* infection did not significantly alter PRDX1 fluorescence intensity (Supplementary Figure S7E).

**Figure 8:**
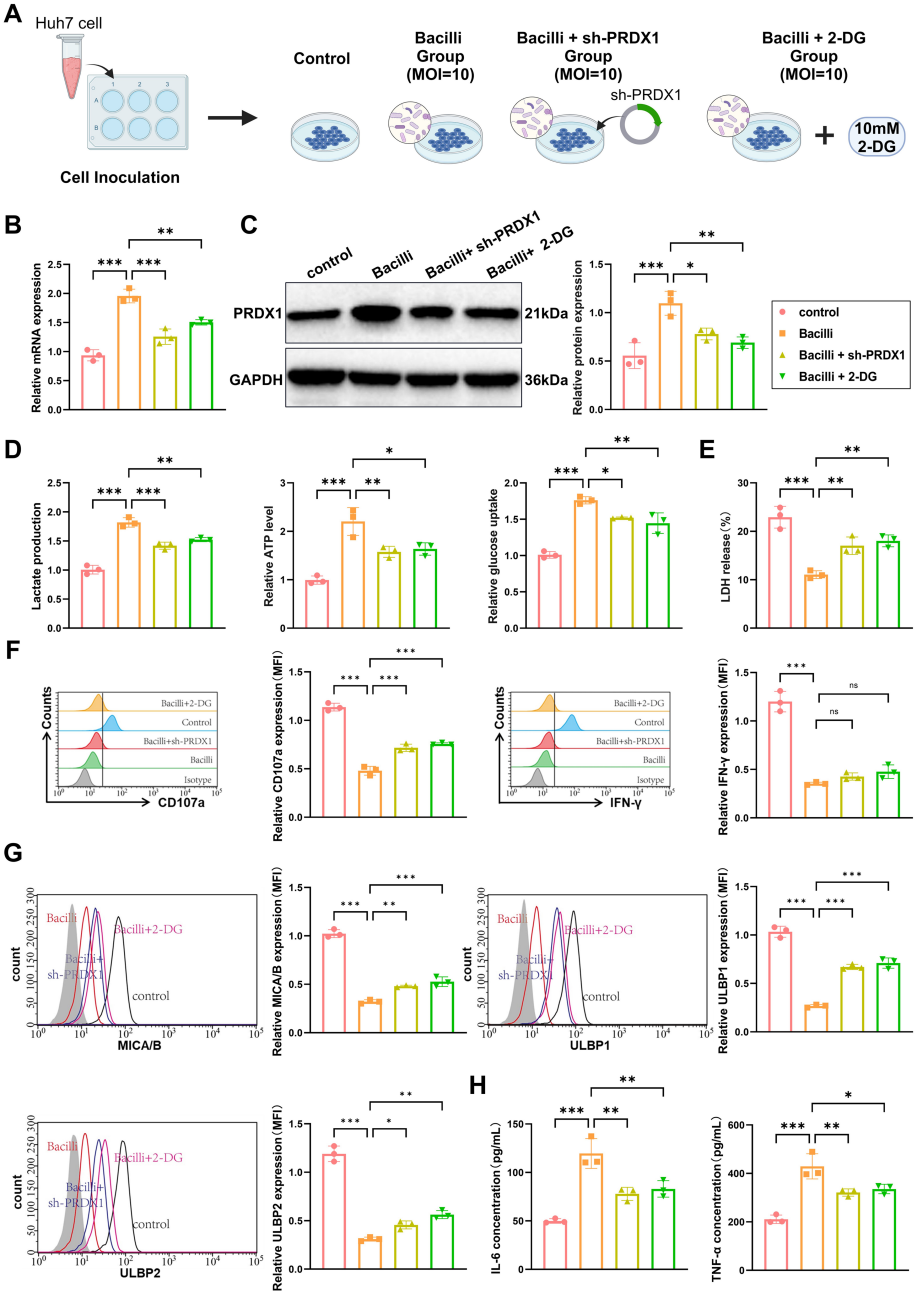
Bacilli infection promotes immune evasion in liver cancer by upregulating PRDX1 and activating glycolytic pathways. **(A)** Experimental workflow diagram. **(B)** RT-qPCR and Western blot analysis of PRDX1 mRNA and protein expression in Huh7 cells. **(C)** Lactic acid assay and ATP assay measuring lactate concentration in Huh7 cell culture supernatant and intracellular ATP levels, respectively. **(D)** Glucose uptake measured using a glucose assay kit in Huh7 cells. **(E)** LDH release assay to determine Huh7 cell lysis rate. **(F)** Flow cytometry analysis of NK cell activation markers CD107a and IFN-γ. **(G)** Flow cytometry analysis of NKG2D ligands (MICA/B, ULBP1/2) expression on the surface of Huh7 cells. **(H)** ELISA to measure the concentrations of IL-6 and TNF-α in the co-culture supernatant. All cell experiments were performed in triplicate, with * indicating *p* < 0.05, ** indicating *p* < 0.01, and *** indicating *p* < 0.001.

To investigate whether *Bacilli* infection promotes immune evasion via PRDX1 upregulation and activation of glycolytic pathways, we established four experimental groups: (1) Control: uninfected Huh7 cells; (2) Bacilli: Huh7 cells infected with *Bacilli* (MOI = 10); (3) Bacilli + sh-PRDX1: *Bacilli*-infected cells with PRDX1 knockdown; (4) Bacilli + 2-DG: *Bacilli*-infected cells treated with 2-deoxy-D-glucose (2-DG, 10 mM), a glycolysis inhibitor (Figure 8A).

RT-qPCR and western blot analyses confirmed that PRDX1 expression was significantly elevated in the *Bacilli* group and was partially reversed in the sh-PRDX1 group and the Bacilli + 2-DG group (Figure 8B). Measurements of lactate secretion and intracellular ATP levels revealed significant increases in the *Bacilli* group, both of which were partially reversed in the Bacilli + sh-PRDX1 and Bacilli + 2-DG groups. Glucose uptake followed a similar pattern, being enhanced by *Bacilli* infection and attenuated in the Bacilli + sh-PRDX1 and Bacilli + 2-DG groups (Figure 8C).

To assess NK cell cytotoxicity, activated NK cells were co-cultured with Huh7 cells from the various treatment groups at an effector-to-target (E:T) ratio of 1:10 for 12 h. An LDH release assay revealed a marked reduction in NK cell-mediated lysis in the *Bacilli* group (Figure 8D). Flow cytometry analysis using anti-CD107a and anti-IFN-γ antibodies showed decreased expression of both markers in the *Bacilli*-infected cells (Figure 8E). Additionally, *Bacilli* infection significantly downregulated NKG2D ligands (MICA/B and ULBP1/2) on Huh7 cells, as assessed by flow cytometry (Figure 8G). These immunosuppressive effects were partially alleviated in the Bacilli + sh-PRDX1 and Bacilli + 2-DG groups (Figures 8D–F).

Cytokine analysis using ELISA indicated that secretion of IL-6 and TNF-*α* was significantly increased in the *Bacilli* group, whereas both cytokines were significantly reduced in the Bacilli + sh-PRDX1 and Bacilli + 2-DG groups (Figure 8H).

Collectively, these findings demonstrate that *Bacilli* infection promotes immune evasion in HCC cells by upregulating PRDX1, which in turn activates glycolytic pathways. This metabolic reprogramming suppresses NK cell cytotoxicity and downregulates NKG2D ligands, facilitating tumor immune evasion.

### *In vivo* validation of *Bacilli* infection-induced PRDX1 upregulation and glycolytic activation driving immune evasion in HCC

**Figure 9:**
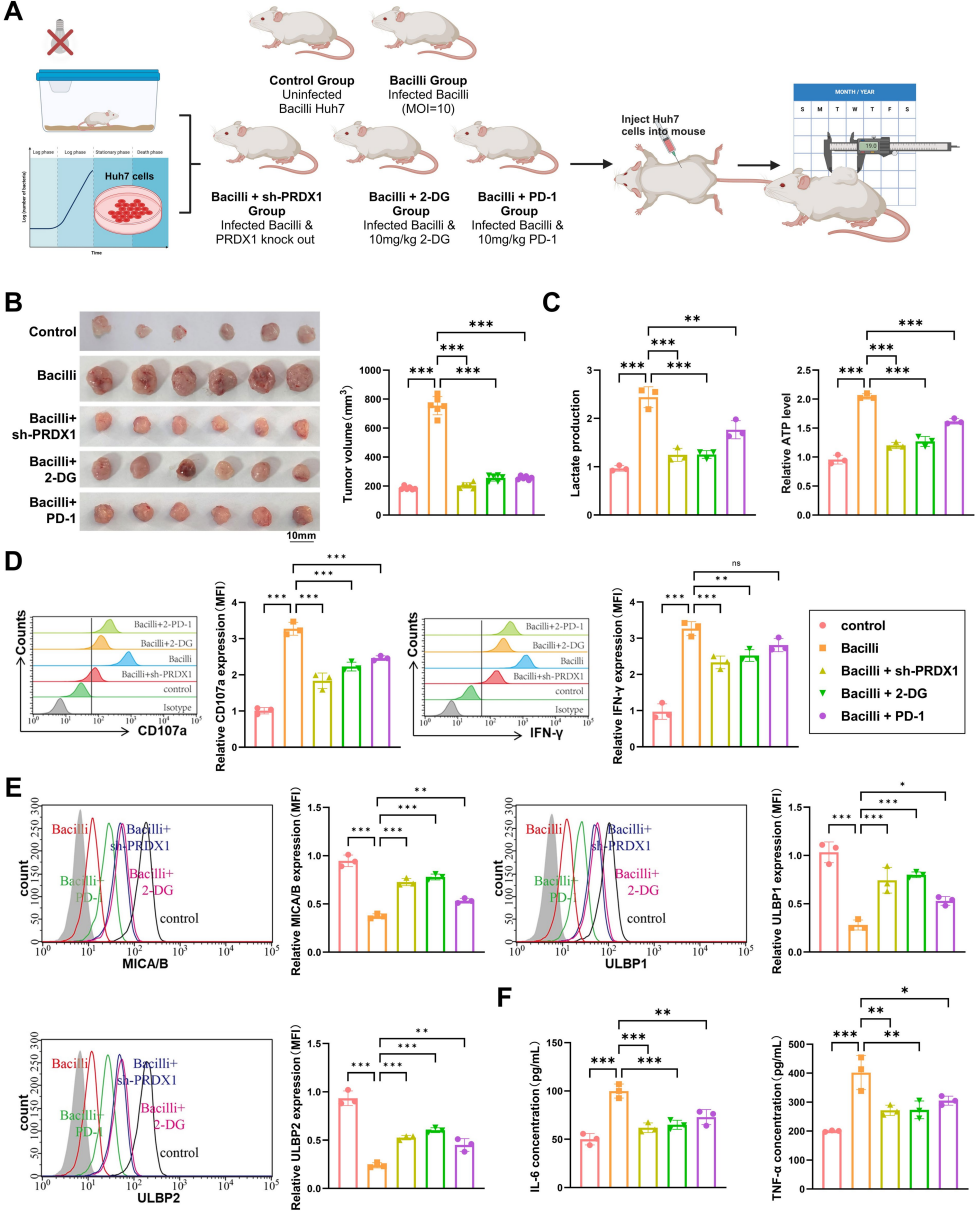
Bacilli infection alters tumor growth, metabolism, immune microenvironment, and glycolytic markers in a nude mouse xenograft model. **(A)** Animal model and experimental workflow diagram. **(B)** Tumor volume monitoring showing differences in tumor size between treatment groups. **(C)** Lactic acid and ATP assays to measure lactate concentration and ATP levels in tumor tissue. **(D)** Flow cytometry analysis of NK cell activation markers (CD107a and IFN-γ). **(E)** Flow cytometry analysis of NKG2D ligands (MICA/B, ULBP1/2) expression on the surface of tumor cells. **(F)** ELISA to assess the levels of IL-6 and TNF-α in the tumor microenvironment. Animal experiments were conducted with *N* = 6, with * indicating *p* < 0.05, ** indicating *p* < 0.01, and *** indicating *p* < 0.001.

To validate the role of *Bacilli* infection in promoting tumor progression and immune evasion via PRDX1-mediated glycolytic activation, an *in vivo* xenograft model was established using Huh7 cells in nude mice. The mice were divided into five groups: Control (uninfected), *Bacilli* infection (Bacilli), *Bacilli* infection with PRDX1 knockdown (Bacilli + sh-PRDX1), *Bacilli* infection with glycolysis inhibition (Bacilli + 2-DG), and *Bacilli* infection with PD-1 blockade (Bacilli + PD-1). The experimental design is summarized in Figure 9A.

Tumor growth was monitored over time. The *Bacilli*-infected group exhibited a significant increase in tumor volume compared to the Control group. Notably, tumor growth was markedly suppressed in the Bacilli + sh-PRDX1, Bacilli + 2-DG, and Bacilli + PD-1 groups, with final tumor volumes comparable to those observed in the Control group (Figure 9B).

Metabolic profiling revealed that lactate production and intracellular ATP levels were significantly elevated in tumor tissues from the *Bacilli* group, indicating enhanced glycolytic activity. These elevations were substantially reversed in the Bacilli + sh-PRDX1 and Bacilli + 2-DG groups, while the Bacilli + PD-1 group showed partial restoration toward baseline levels (Figure 9C).

Flow cytometry analysis of intratumoral immune markers revealed a significant reduction in NK cell activation in the *Bacilli* group, as evidenced by decreased expression of CD107a and IFN-γ. These markers were significantly restored in the Bacilli + sh-PRDX1 and Bacilli + 2-DG groups, and partially restored in the Bacilli + PD-1 group (Figure 9D).

Consistent with these findings, expression of the NK cell-activating ligands MICA/B and ULBP1/2 on tumor cells was markedly downregulated in the *Bacilli* group. However, expression was substantially restored in the Bacilli + sh-PRDX1 and Bacilli + 2-DG groups, with partial improvement in the Bacilli + PD-1 group (Figure 9E).

ELISA assays performed on tumor-associated immune cell supernatants revealed that IL-6 and TNF-α secretion was significantly increased in the *Bacilli* group. These proinflammatory cytokines were significantly reduced in the Bacilli + sh-PRDX1 and Bacilli + 2-DG groups, and partially suppressed in the Bacilli + PD-1 group (Figure 9F).

Collectively, these *in vivo* findings confirm that *Bacilli* infection enhances PRDX1 expression and glycolytic reprogramming in HCC, leading to suppression of NK cell-mediated cytotoxicity and promoting tumor immune evasion. Targeting PRDX1, inhibiting glycolysis, or applying immune checkpoint blockade (anti-PD-1) can effectively counteract these immunosuppressive effects and restore antitumor immune activity.

## Discussion

With advances in cancer biology, growing attention has been directed toward the role of the TME—particularly the microbial component—in influencing tumor progression and therapeutic outcomes (Yu et al., 2024; Sepich-Poore et al., 2021). HCC, a leading cause of cancer-related mortality worldwide (Liu H. et al., 2024; Liu P. et al., 2024; Kudo et al., 2025), presents substantial challenges in immunotherapy efficacy. These challenges are multifactorial, with emerging evidence suggesting a critical yet incompletely understood role of the microbiome in modulating immune responses (Zhang et al., 2024; Jin et al., 2025). While most previous studies have primarily focused on the link between gut microbiota and tumor initiation or progression (Huo et al., 2024), our study advances the field by integrating single-cell multi-omics data to elucidate how specific microbial taxa influence immunotherapy responses in HCC through metabolic pathways, particularly glycolysis. This novel approach offers critical insights into the dynamic crosstalk between the microbiota and tumor metabolism, providing a foundation for more personalized and precise therapeutic strategies.

In our analysis comparing the gut microbiota of HCC patients and healthy controls, we identified a significant enrichment of *Bacilli*, *Lactobacillales*, and *Streptococcaceae* in HCC patients. This microbial signature appears distinct from those reported in other malignancies, suggesting a tumor-type-specific microbiome profile. Prior studies have largely focused on the impact of individual bacterial species on tumor biology (Aslam et al., 2024), demonstrating that certain microbes can enhance antitumor immunity by promoting the recruitment and activation of dendritic cells, CD8^+^ T cells, and NK cells, thereby augmenting the efficacy of immune checkpoint inhibitors (Yang C. et al., 2024; Yang L. et al., 2024). In contrast, our study shifts the focus from individual species to the broader functional consequences of microbial community shifts, particularly how these changes influence immune evasion mechanisms in the liver TME. This distinction underscores the importance of considering patient-specific microbial alterations when designing immunotherapeutic strategies for HCC.

A central finding of this study is the role of the glycolytic pathway in mediating immune evasion in HCC, with PRDX1 emerging as a key regulatory node. We observed a marked upregulation of PRDX1 in response to *Bacilli* infection, along with enhanced glycolytic activity. PPI network analysis revealed that PRDX1 is closely connected to other glycolysis-related proteins, suggesting a central role in metabolic reprogramming. Unlike prior research that has largely emphasized cytokine signaling or general inflammatory responses, our study probes deeper into how metabolic alterations at the cellular level directly affect immune cell function—particularly NK cell surveillance. The growing recognition of the TME’s complexity, including the influence of infiltrating immune and stromal cells, has underscored its significance in determining clinical outcomes in malignant tumors (Wang et al., 2022a, 2022b). Our findings contribute to this understanding by providing new evidence that links metabolic reprogramming—specifically glycolysis—to the suppression of antitumor immunity, highlighting a previously underappreciated axis of immune evasion regulated by microbial-metabolic interactions.

To reinforce the logical foundation of our multi-omics approach, we adopted a stepwise mechanistic strategy that traces the progression from gut microbiota alterations to metabolic reprogramming, immune modulation, and ultimately the identification of core regulatory factors. We first used 16S rRNA sequencing data to identify characteristic microbial taxa enriched in HCC patients. Functional prediction using PICRUSt and enrichment analysis revealed that these taxa were strongly associated with glycolysis and other metabolic pathways. Next, we analyzed scRNA-seq data and observed immune-suppressive features within the HCC microenvironment, including reduced NK cell infiltration and impaired intercellular communication—changes potentially driven by microbiota-associated metabolic rewiring. Building on these findings, we integrated transcriptomic data from the TCGA-LIHC cohort and performed differential expression analysis, PPI network construction, and pathway enrichment intersection. This integrative analysis identified PRDX1 as a key regulatory gene that links glycolysis with immune modulation. This “microbiota-metabolism-immunity-core gene” framework offers a coherent and biologically grounded model to explain the interplay between microbial composition and tumor immune evasion, and it sets the stage for future validation using matched multi-omics datasets.

By analyzing gene expression alterations induced by bacterial infection in hepatocytes through single-cell transcriptomics, our study achieves a high-resolution view of cellular heterogeneity and uncovers how bacterial influences reshape tumor cell metabolism and immune evasion mechanisms. Unlike traditional bulk transcriptomic or proteomic analyses, which average signals across diverse cell types, this single-cell approach enables precise dissection of cell-type-specific responses and allows for the identification and mechanistic validation of key modulators such as PRDX1.

Given the shared metabolic pathways between tumor cells and immune cells, targeting tumor metabolism can unintentionally affect immune function. Therefore, understanding how tumor metabolic reprogramming modulates the immune microenvironment is essential for the development of therapeutic strategies that maximize antitumor efficacy while preserving immune cell activity. Achieving this balance is crucial, as it may open new avenues for metabolic targeting within the TME (Song et al., 2025). Our findings show that bacterial infection activates glycolysis and the NF-κB signaling pathway, significantly impairing the efficacy of anti-PD-1 therapy. This provides clear evidence that microbial signals can modulate immunotherapy outcomes through both metabolic and inflammatory mechanisms. These insights underscore the potential clinical relevance of manipulating microbiota or targeting specific metabolic pathways to enhance the therapeutic benefit of immune checkpoint inhibitors in HCC.

From the perspective of microbiome-metabolism-immune interactions, this is the first study to reveal the role of the gut microbiota-PRDX1-glycolysis axis in mediating immune evasion in HCC. We propose a novel mechanism by which gut microbiota influence both tumor metabolism and immunity. The upregulation of PRDX1 and enhanced glycolytic activity provide not only the energy required for tumor proliferation but also immunosuppressive signals that facilitate tumor immune evasion. These results identify PRDX1 as a critical mediator bridging glycolytic metabolism and immune suppression, thereby offering new insights into HCC pathophysiology. Moreover, targeting PRDX1 or modulating the gut microbiota may represent promising strategies for improving the effectiveness of immunotherapy and developing precision treatments for HCC.

This study has several limitations that warrant consideration. First, it primarily focuses on *Bacilli* and does not fully capture the complexity and diversity of the gut microbiota in HCC. Second, while PRDX1 was identified as a key regulator of glycolysis and immune modulation, the precise molecular mechanisms through which PRDX1 governs glycolytic flux and immune suppression were not comprehensively dissected. Furthermore, the study does not evaluate the universality of PRDX1 expression or function across various HCC subtypes or disease stages. Additionally, the long-term efficacy and combinatorial potential of PD-1 immune checkpoint inhibitors in the context of *Bacilli*-induced metabolic reprogramming require further investigation. Given the distinct origins of the 16S and scRNA-seq datasets, this study proposes only a mechanistic hypothesis based on indirect evidence, and future studies integrating matched multi-omics data from the same cohort are warranted for validation. Expanding the sample size and incorporating advanced multi-omics platforms, including metabolomics and proteomics, alongside single-cell analyses will allow for a more detailed exploration of PRDX1’s regulatory network and the broader immunometabolic impact of the gut microbiota. Moreover, therapeutic strategies combining PRDX1 inhibitors, glycolytic pathway inhibitors, and immune checkpoint blockade should be systematically evaluated. Innovative microbiome-based therapies, such as probiotics or fecal microbiota transplantation, also hold promise for modulating the TIME and improving clinical outcomes in HCC.

To further contextualize our findings, it is important to acknowledge previous studies that have explored similar immune-metabolic regulatory mechanisms or microbial involvement in tumor progression. For example, Li et al. reported that *Fusobacterium nucleatum* promotes oral squamous cell carcinoma proliferation via the E-cadherin/β-catenin pathway (Li et al., 2024), highlighting the broader role of bacteria in modulating cancer signaling. Similarly, Wan et al. (2024) and Wang et al. (2024) applied multi-omics and machine learning approaches to dissect immune-related signatures and metabolic pathways in gastric and brain cancers, respectively. These works support our strategy of leveraging multi-omics data to identify immunotherapeutic targets. Moreover, recent single-cell analyses by Ye et al. (2023) and Zhang et al. (2023) revealed distinct tumor cell subpopulations linked to clinical prognosis across pancreatic and breast cancers. Finally, Li et al. (2023) demonstrated the prognostic relevance of ferroptosis-related genes in head and neck squamous cell carcinoma using a similar multi-omics pipeline. These studies collectively underscore the importance and generalizability of microbiota- and metabolism-centered frameworks in understanding tumor immune evasion. Our study builds upon and differentiates from these efforts by specifically elucidating the PRDX1-driven glycolytic axis in HCC.

## Conclusion

Through integrative multi-omics analysis, this study reveals a novel mechanism by which the gut microbiota—particularly *Bacilli*—contributes to immune evasion in HCC. We demonstrate that *Bacilli* infection leads to the upregulation of PRDX1, which activates glycolytic pathways and suppresses NK cell activity, thereby facilitating tumor immune evasion. Comparative microbiota profiling showed a significant enrichment of *Bacilli* and *Streptococcaceae* in HCC patients relative to healthy controls. Functional prediction indicated that microbial metabolic products influence both glycolytic metabolism and the immune microenvironment. PRDX1 was found to be markedly upregulated in both HCC patient samples and *Bacilli*-infected tumor models. Its expression correlates with elevated levels of key glycolysis-associated genes, including HIF-1α, LDHA, and PKM2, leading to increased lactate accumulation and impaired NK cell cytotoxicity. Functional validation using *in vitro* and *in vivo* models confirmed that PRDX1 knockdown or pharmacological inhibition of glycolysis via 2-DG effectively restored NK cell-mediated antitumor activity and reversed immune suppression. Moreover, PD-1 immune checkpoint blockade showed partial restoration of immune function within the infection model. In summary, this study identifies PRDX1 as a critical mediator of immune evasion in HCC via the glycolytic pathway, acting downstream of specific microbial stimuli. These findings underscore the importance of tumor-microbiota-metabolism crosstalk in shaping immune responses and highlight PRDX1 and glycolysis as promising targets for therapeutic intervention. Furthermore, modulation of the gut microbiota may represent a novel and complementary strategy for enhancing the efficacy of immunotherapy in HCC (graphical abstract).

## Data Availability

Relevant datasets were identified in the EMBL-EBI database (https://www.ebi.ac.uk/ena/browser/search) using the keyword “Liver cancer.” Phenotypic information for all samples within the selected project (BioProject ID: PRJNA1127013) was retrieved. The study included 17 HCC samples (tumors graded as stage III or above) and 12 healthy control samples. Corresponding 16S rRNA sequencing data were downloaded from the NCBI Sequence Read Archive (SRA) database (https://www.ncbi.nlm.nih.gov/sra/). The full reproducible code used to analyze public datasets (PRJNA1127013 and GSE189903) is provided in the Supplementary Code Files 1, 2.

## Footnotes

^1^ https://www.ebi.ac.uk/ena/browser/search

^2^ https://www.ncbi.nlm.nih.gov/sra/

^3^ https://huttenhower.sph.harvard.edu/kneaddata/

^4^ http://www.loucalab.com/archive/FAPROTAX/

^5^ http://xteam.xbio.top/CellMarker/

^6^ https://www.kegg.jp/kegg/rest/keggapi.html

^7^ https://www.genecards.org/

^8^ https://string-db.org/

^9^ https://portal.gdc.cancer.gov

^10^ http://bio-computing.hrbmu.edu.cn/gutmgene/#/home

^11^ https://www.metaboanalyst.ca/

## Supplementary material

SUPPLEMENTARY FIGURE S1 Analysis of gut microbiota species diversity in fecal samples from HCC patients and healthy individuals. **(A)** Rarefaction curve of gut microbiota α-diversity in the HCC and Healthy groups. **(B–G)** Alpha diversity analysis of gut microbiota in the HCC and Healthy groups. **(B)** Shows the Chao1 index. **(C)** Shows the Invsimpson index. **(D)** Shows the Richness index. **(E)** Shows the Shannon index. **(F)** Shows the Simpson index. **(G)** Shows the ACE index. **(H)** Beta diversity analysis of gut microbiota in the HCC and Healthy groups using PCoA. **(I)** Manhattan plot comparing abundance differences between the Healthy and HCC groups. **(J)** Stacked bar chart showing the relative abundance of gut microbiota at the phylum level, with different colors representing different phyla; Healthy: *n* = 12; HCC: *n* = 17.

SUPPLEMENTARY FIGURE S2 Schematic overview of multi-omics integration and experimental validation strategy. This diagram illustrates the integrative workflow of 16S rRNA sequencing and scRNA-seq analyses used to uncover microbiota-driven immunometabolic alterations in HCC. Signature microbiota were identified from public 16S datasets and linked to glycolysis-related metabolic pathways via PICRUSt analysis. Concurrently, scRNA-seq data from HCC samples revealed immunosuppressive features and impaired intercellular communication using Seurat analysis. Combined with TCGA transcriptomic data and PPI network analysis, PRDX1 was identified as a key glycolysis-associated immunoregulatory target.

SUPPLEMENTARY FIGURE S3 Quality control, filtering, and PCA of scRNA-seq data. **(A)** Violin plots of gene count (nFeature_RNA), mRNA molecule count (nCount_RNA), and mitochondrial gene percentage (percent.mt) for each cell in the scRNA-seq data. **(B)** Correlation scatter plots between filtered data for nCount_RNA vs. percent.mt, and nCount_RNA vs. nFeature_RNA. **(C)** Heatmap of the top 20 genes most strongly correlated with PC_1-PC_6 in PCA, where yellow indicates upregulated expression and purple indicates downregulated expression. **(D)** Distribution of cells in PC_1 and PC_2 before batch correction (left), with each point representing a cell, and violin plots of the distribution in PC_1 and PC_2 (right). **(E)** Batch correction process using Harmony, where the x-axis represents the number of interaction iterations. **(F)** Standard deviation distribution of PCs, with more important PCs showing larger standard deviations. **(G)** Distribution of cells in PC_1 and PC_2 after Harmony batch correction (left), with each point representing a cell, and violin plots of the corrected distribution (right); Normal: *n* = 3; HCC: *n* = 4.

SUPPLEMENTARY FIGURE S4 Cell clustering analysis of scRNA-seq data. **(A)** Clustering results at different resolutions using the Clustree package. **(B)** UMAP visualization of clustering results, showing cell aggregation and distribution, where each color represents a distinct cluster. **(C)** UMAP clustering results visualized in two dimensions, displaying cell aggregation and distribution for Normal and HCC samples. Blue represents Normal samples, and red represents HCC samples. Normal: *n* = 3; HCC: *n* = 4.

SUPPLEMENTARY FIGURE S5 GO functional enrichment analysis of DEGs. **(A)** BP analysis. **(B)** CC analysis. **(C)** MF analysis.

SUPPLEMENTARY FIGURE S6 Verification of PRDX1 overexpression and knockdown efficiency. **(A)** RT-qPCR analysis of PRDX1 mRNA expression levels in Huh7 cells for the overexpression, knockdown, and control groups. **(B)** Western blot analysis of PRDX1 protein expression levels in Huh7 cells for the overexpression, knockdown, and control groups. All cell experiments were triplicate, with ** indicating *p* < 0.01 and *** indicating *p* < 0.001.

SUPPLEMENTARY FIGURE S7 Effects of specific bacterial infections on PRDX1 expression and immune-related molecules in HCC cells. **(A)** Experimental workflow diagram. **(B)** RT-qPCR analysis of the effect of Bacilli and Streptococcaceae infections on PRDX1 mRNA expression in Huh7 cells. **(C)** Western blot analysis of the effect of Bacilli and Streptococcaceae infections on PRDX1 protein expression in Huh7 cells. **(D)** Flow cytometry analysis of the effect of Bacilli and Streptococcaceae infections on the surface expression of NKG2D ligands MICA/B and ULBP1/2 in Huh7 cells, measured by MFI. **(E)** Immunofluorescence staining to assess the effect of Bacilli and Streptococcaceae infections on PRDX1 protein localization and fluorescence intensity in Huh7 cells. All cell experiments were triplicate, with ** indicating *p* < 0.01 and *** indicating *p* < 0.001.

## Glossary

ATCC: American Type Culture Collection
BP: Biological process
CC: Cellular component
DEGs: Differentially expressed genes
FBS: Fetal bovine serum
FDR: False discovery rate
GO: Gene Ontology
HCC: Hepatocellular carcinoma
LDA: Linear discriminant analysis
LEfSe: Linear Discriminant Analysis Effect Size
LDH: Lactate dehydrogenase
MFI: Mean fluorescence intensity
MF: Molecular function
MOI: Multiplicity of infection
NK: Natural killer
OD: Optical density
PCA: Principal component analysis
PCoA: Principal coordinate analysis
PPI: Protein–protein interaction
PRDX1: Peroxiredoxin 1
PBMC: Peripheral blood mononuclear cell
TAMs: Tumor-associated macrophages
UMAP: Uniform Manifold Approximation and Projection
OTUs: Operational taxonomic units
SRA: Sequence Read Archive
