# Supplementary figures and images for: Tumor-associated bacteria activate PRDX1-driven glycolysis to promote immune evasion and PD-1 antibody resistance in hepatocellular carcinoma
Source: Front Microbiol. 2025 Jul 7;16:1599691. doi: 10.3389/fmicb.2025.1599691 (PMC12277338; doi:10.3389/fmicb.2025.1599691)

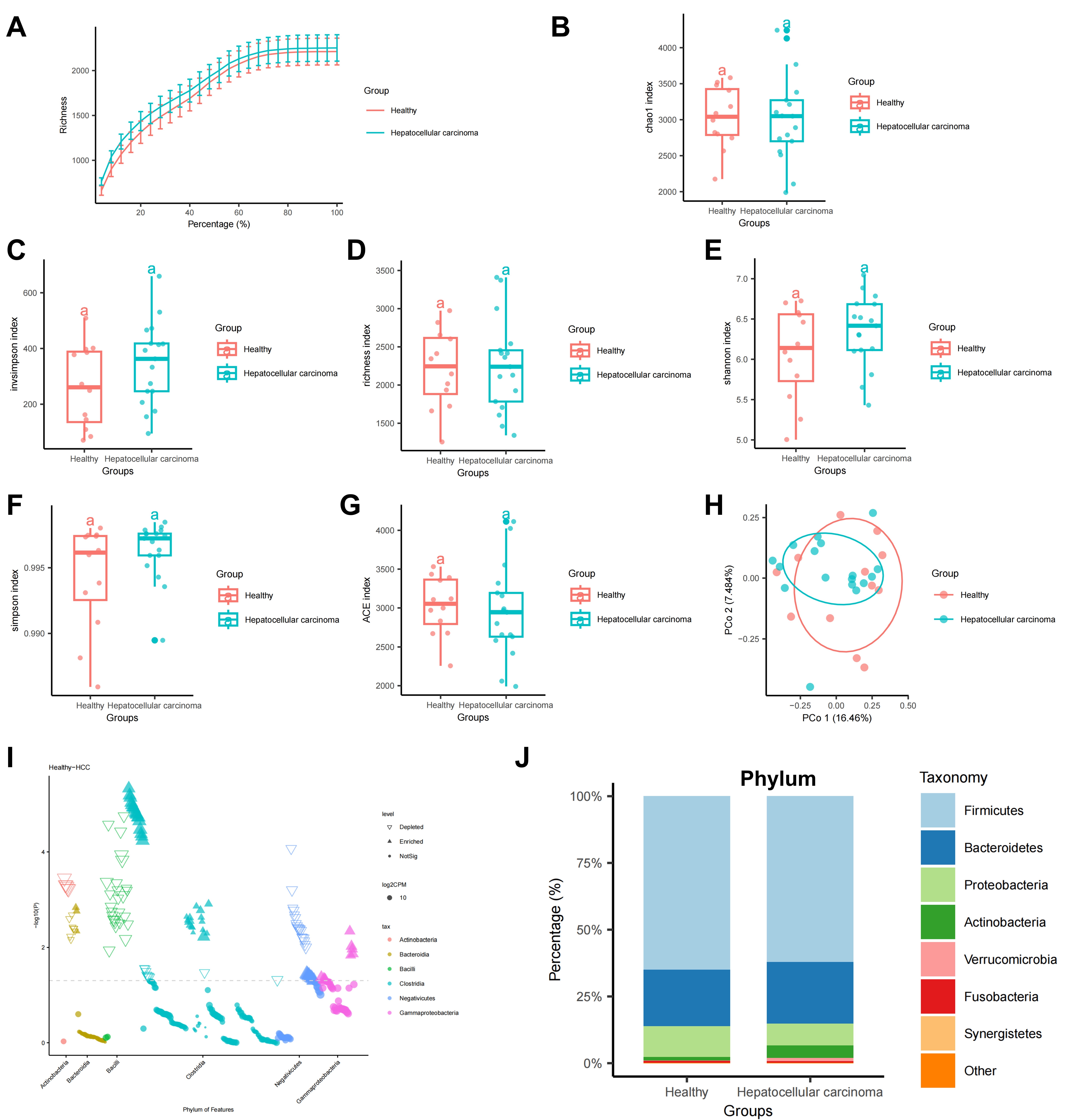

Supplement: SUPPLEMENTARY FIGURE S1 — Analysis of gut microbiota species diversity in fecal samples from HCC patients and healthy individuals. (A) Rarefaction curve of gut microbiota α-diversity in the HCC and Healthy groups. (B–G) Alpha diversity analysis of gut microbiota in the HCC and Healthy groups. (B) Shows the Chao1 index. (C) Shows the Invsimpson index. (D) Shows the Richness index. (E) Shows the Shannon index. (F) Shows the Simpson index. (G) Shows the ACE index. (H) Beta diversity analysis of gut microbiota in the HCC and Healthy groups using PCoA. (I) Manhattan plot comparing abundance differences between the Healthy and HCC groups. (J) Stacked bar chart showing the relative abundance of gut microbiota at the phylum level, with different colors representing different phyla; Healthy: n = 12; HCC: n = 17. [file Image_1.jpeg]

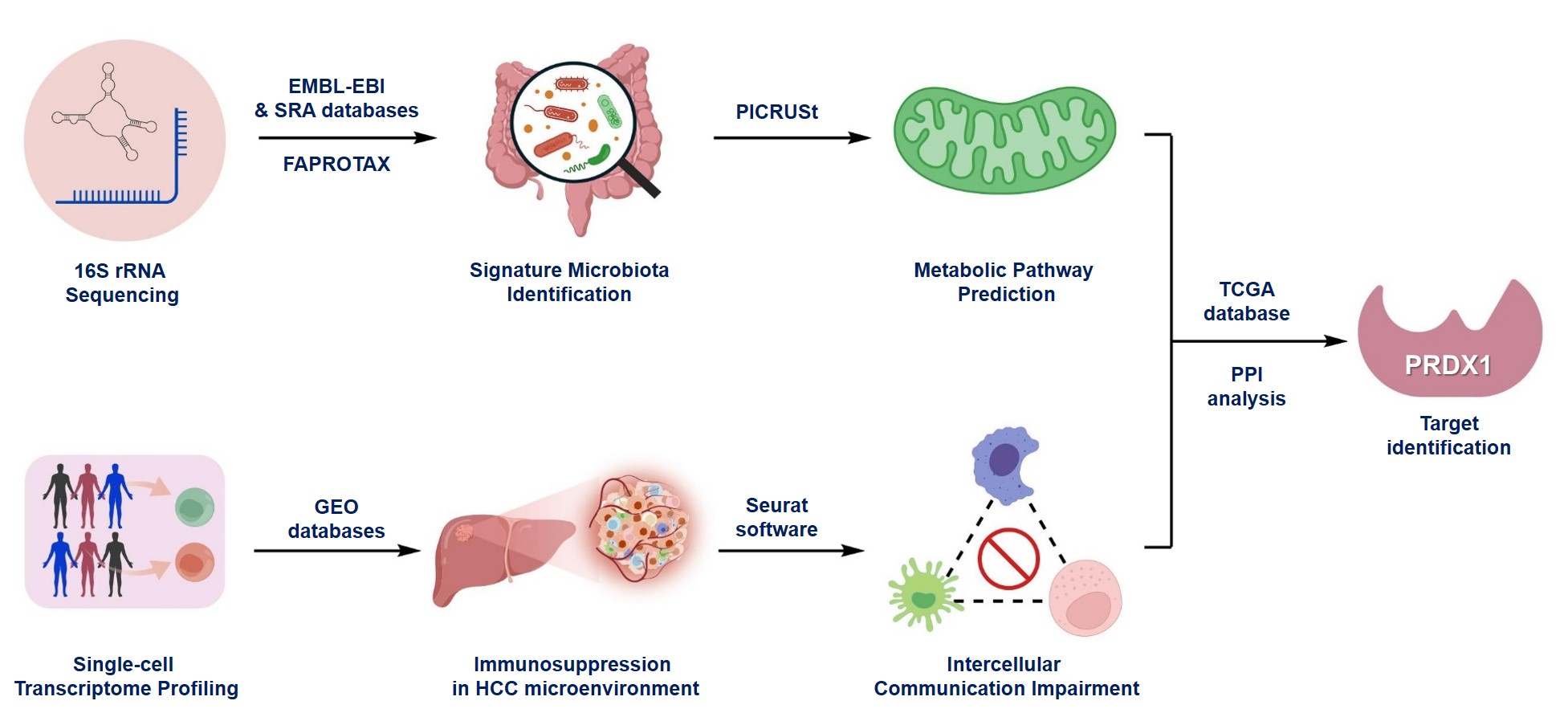

Supplement: SUPPLEMENTARY FIGURE S2 — Schematic overview of multi-omics integration and experimental validation strategy. This diagram illustrates the integrative workflow of 16S rRNA sequencing and scRNA-seq analyses used to uncover microbiota-driven immunometabolic alterations in HCC. Signature microbiota were identified from public 16S datasets and linked to glycolysis-related metabolic pathways via PICRUSt analysis. Concurrently, scRNA-seq data from HCC samples revealed immunosuppressive features and impaired intercellular communication using Seurat analysis. Combined with TCGA transcriptomic data and PPI network analysis, PRDX1 was identified as a key glycolysis-associated immunoregulatory target. [file Image_2.jpeg]

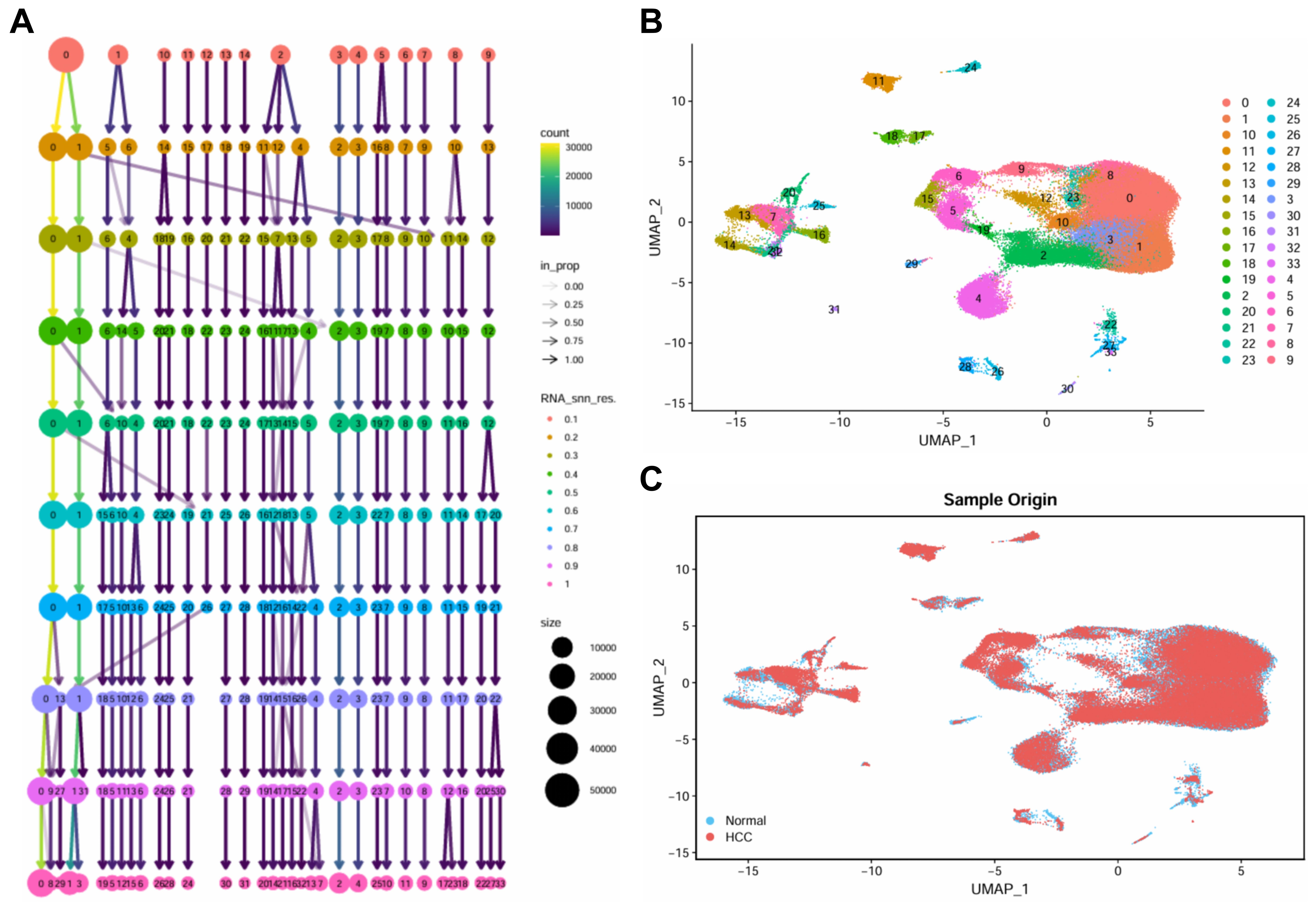

Supplement: SUPPLEMENTARY FIGURE S4 — Cell clustering analysis of scRNA-seq data. (A) Clustering results at different resolutions using the Clustree package. (B) UMAP visualization of clustering results, showing cell aggregation and distribution, where each color represents a distinct cluster. (C) UMAP clustering results visualized in two dimensions, displaying cell aggregation and distribution for Normal and HCC samples. Blue represents Normal samples, and red represents HCC samples. Normal: n = 3; HCC: n = 4. [file Image_4.jpeg]

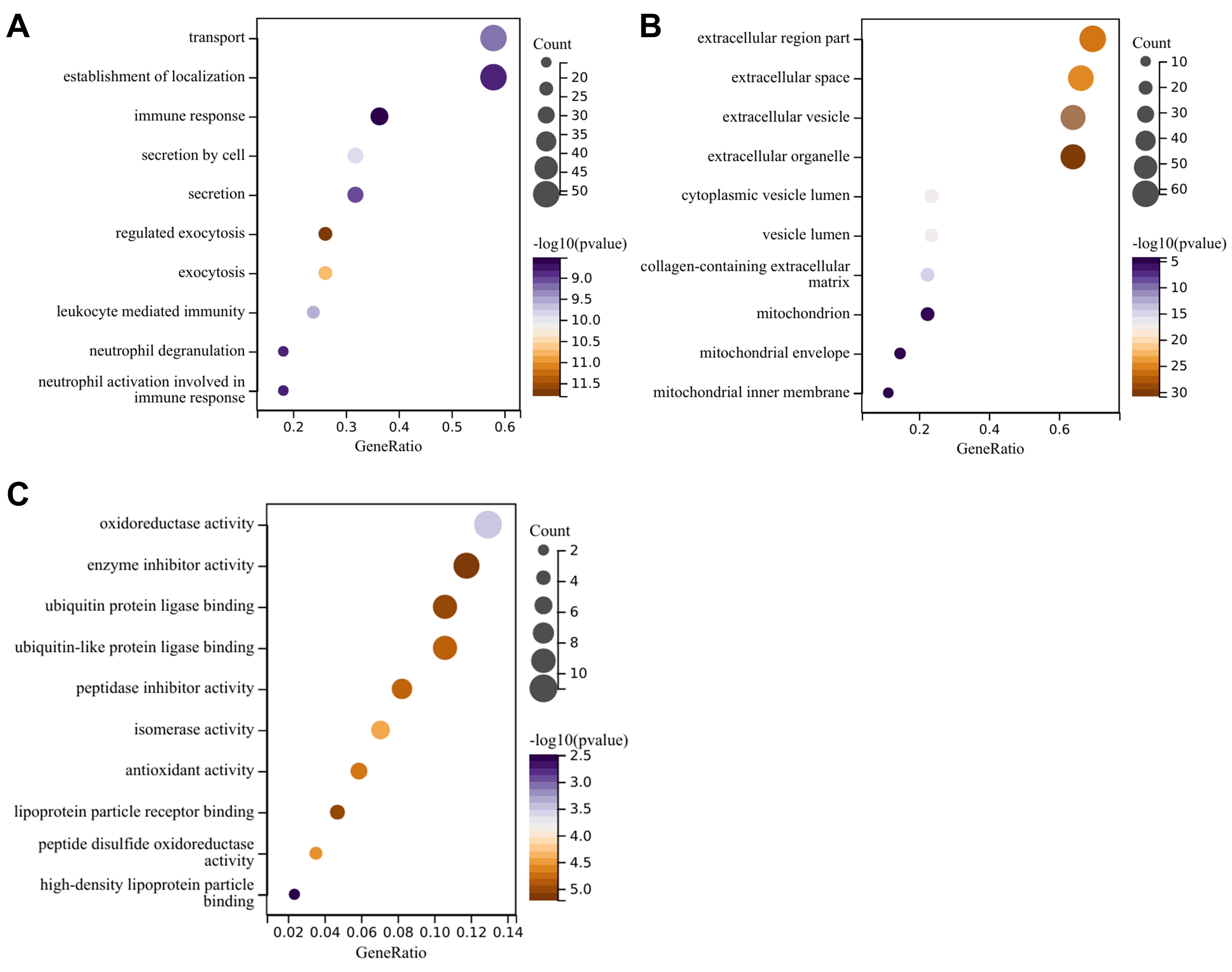

Supplement: SUPPLEMENTARY FIGURE S5 — GO functional enrichment analysis of DEGs. (A) BP analysis. (B) CC analysis. (C) MF analysis. [file Image_5.jpeg]

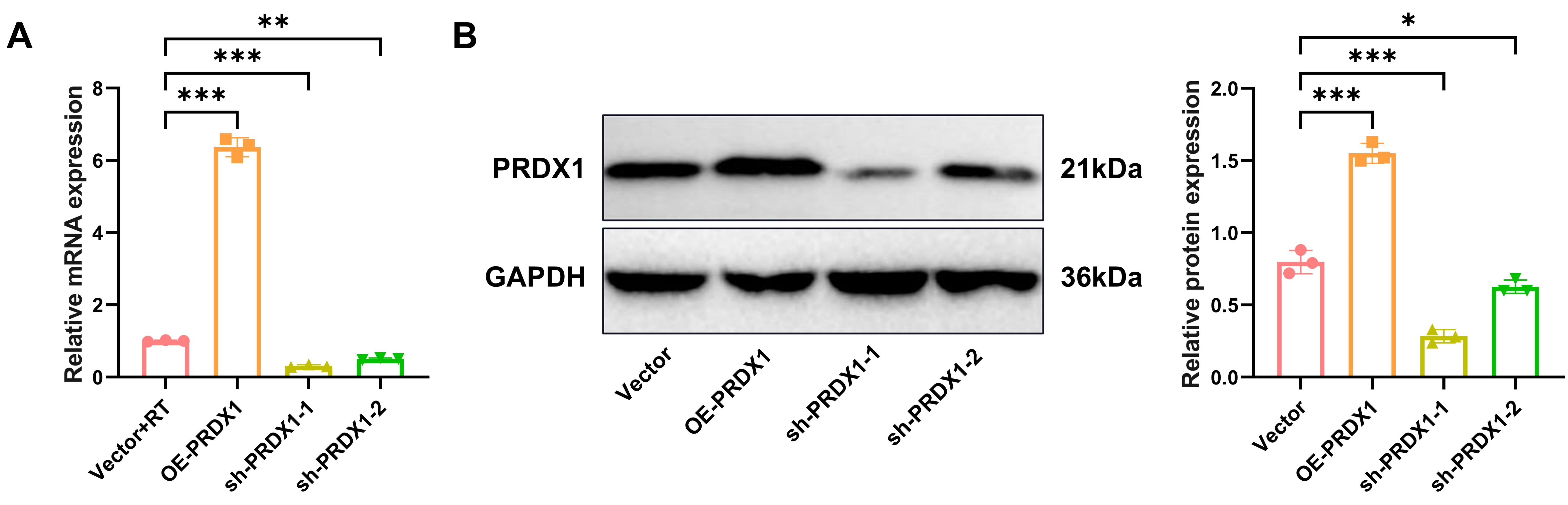

Supplement: SUPPLEMENTARY FIGURE S6 — Verification of PRDX1 overexpression and knockdown efficiency. (A) RT-qPCR analysis of PRDX1 mRNA expression levels in Huh7 cells for the overexpression, knockdown, and control groups. (B) Western blot analysis of PRDX1 protein expression levels in Huh7 cells for the overexpression, knockdown, and control groups. All cell experiments were triplicate, with ** indicating p < 0.01 and *** indicating p < 0.001. [file Image_6.jpeg]

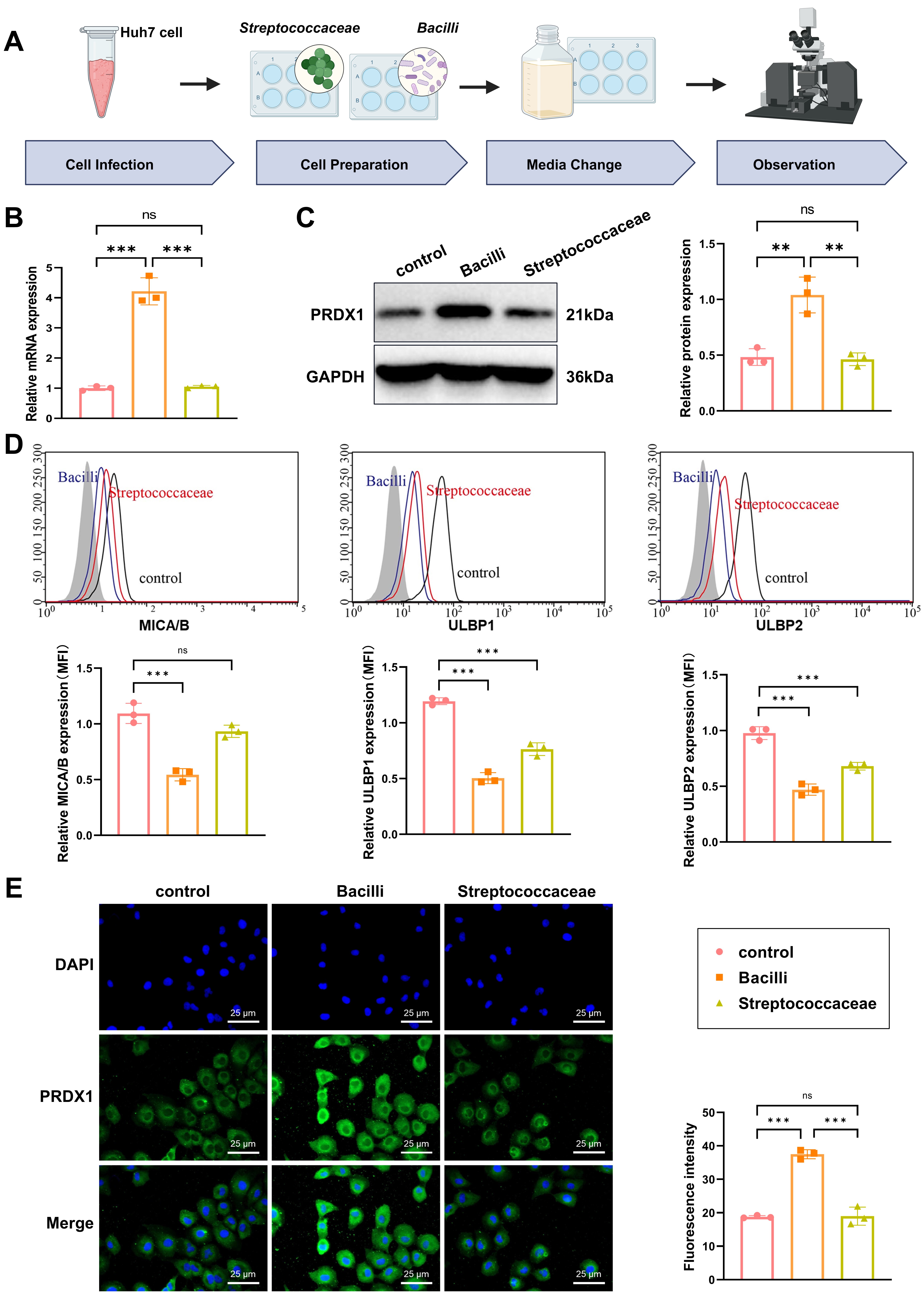

Supplement: SUPPLEMENTARY FIGURE S7 — Effects of specific bacterial infections on PRDX1 expression and immune-related molecules in HCC cells. (A) Experimental workflow diagram. (B) RT-qPCR analysis of the effect of Bacilli and Streptococcaceae infections on PRDX1 mRNA expression in Huh7 cells. (C) Western blot analysis of the effect of Bacilli and Streptococcaceae infections on PRDX1 protein expression in Huh7 cells. (D) Flow cytometry analysis of the effect of Bacilli and Streptococcaceae infections on the surface expression of NKG2D ligands MICA/B and ULBP1/2 in Huh7 cells, measured by MFI. (E) Immunofluorescence staining to assess the effect of Bacilli and Streptococcaceae infections on PRDX1 protein localization and fluorescence intensity in Huh7 cells. All cell experiments were triplicate, with ** indicating p < 0.01 and *** indicating p < 0.001. [file Image_7.jpeg]
