# Supplementary material for: Tumor-associated bacteria activate PRDX1-driven glycolysis to promote immune evasion and PD-1 antibody resistance in hepatocellular carcinoma
Source: Front Microbiol. 2025 Jul 7;16:1599691. doi: 10.3389/fmicb.2025.1599691 (PMC12277338; doi:10.3389/fmicb.2025.1599691)
Supplement: Supplementary file 8 [file Data_Sheet_1.docx]

Supplementary Code 1

Steps:

In this study, we analyzed 16S rRNA sequencing data from the SRA database (Project ID: PRJNA1127013), including fecal samples from 17 hepatocellular carcinoma (HCC) patients and 12 healthy individuals, to characterize gut microbiota composition and diversity. The workflow included the following steps:

1. Data Import and Phyloseq Object Construction

OTU tables (otu_table.txt), taxonomy annotations (taxonomy_table.txt), and metadata (sample_metadata.txt) were loaded.

A phyloseq object was constructed to standardize downstream microbial ecology analysis.

2. Alpha Diversity Analysis

Within-sample microbial diversity was assessed using common alpha diversity indices, including Shannon, Simpson, Chao1, ACE, InvSimpson, and Observed species.

Diversity metrics were visualized to compare groups (Figure S1B–G), revealing no significant difference in species richness.

3. Beta Diversity Analysis

Bray-Curtis dissimilarity matrices were calculated and visualized using Principal Coordinates Analysis (PCoA).

HCC and Healthy samples showed distinct clustering in ordination space (Figure S1H), suggesting differences in microbial community structure.

4. OTU Intersection and Venn Diagram

OTUs present in each group were compared and visualized using a Venn diagram (Figure 1A).

Results showed 66 unique OTUs in the HCC group and 109 unique OTUs in the Healthy group.

5. Differential Abundance Analysis

Using the edgeR package, OTU counts were normalized and tested for group-wise differential abundance.

Volcano and Manhattan plots were generated to highlight significantly enriched or depleted OTUs (Figure 1B, Figure S1I).

57 OTUs were significantly enriched in HCC, and 68 OTUs were significantly depleted.

6. Taxonomic Composition Profiling

OTUs were agglomerated at various taxonomic levels (Phylum, Class, Order, Family, Genus) using tax_glom().

Group-wise microbial composition was visualized using stacked bar plots (Figure S1J, Figure 1C–F), revealing that Bacilli, Lactobacillales, and Streptococcaceae were notably enriched in the HCC group.

7. LEfSe Analysis

Linear Discriminant Analysis Effect Size (LEfSe) was performed to identify taxa with significant differential abundance between HCC and Healthy groups.

Based on LDA score > 2, Bacilli, Lactobacillales, and Streptococcaceae were significantly enriched in HCC samples, while Selenomonadales and Sporomusaceae were enriched in Healthy samples (Figure 2A–B).

Script:

# Load required packages

library(phyloseq) # Microbiome analysis framework

library(ggplot2) # General plotting package

library(vegan) # Ecological diversity analysis

library(VennDiagram) # Venn diagram plotting

library(RColorBrewer) # Color palettes

library(gridExtra) # For multi-panel plots

# Set working directory

setwd("your/project/path") # Replace with your actual path

# ----------------------------

# 1. Read OTU table and metadata

# ----------------------------

otu_data <- read.table("result/otu_table.txt", header = TRUE, row.names = 1, sep = "\t")

sample_info <- read.table("result/sample_group.txt", header = TRUE, sep = "\t", row.names = 1)

# Create phyloseq object

OTU <- otu_table(as.matrix(otu_data), taxa_are_rows = TRUE)

SAM <- sample_data(sample_info)

physeq <- phyloseq(OTU, SAM)

# ----------------------------

# 2. Alpha diversity calculation and boxplots

# ----------------------------

alpha_div <- estimate_richness(physeq, measures = c("Observed", "Shannon", "Simpson", "Chao1", "ACE"))

alpha_div$Group <- sample_info$Group

# Example: plot Shannon index

p_alpha <- ggplot(alpha_div, aes(x = Group, y = Shannon, fill = Group)) +

geom_boxplot() +

geom_jitter(width = 0.2) +

theme_bw() +

labs(title = "Shannon Diversity Index", y = "Shannon", x = "")

# ----------------------------

# 3. Beta diversity: PCoA based on Bray-Curtis distance

# ----------------------------

bray_dist <- distance(physeq, method = "bray")

ordination <- ordinate(physeq, method = "PCoA", distance = bray_dist)

p_beta <- plot_ordination(physeq, ordination, color = "Group") +

geom_point(size = 4) +

theme_bw() +

labs(title = "PCoA - Bray-Curtis Distance")

# ----------------------------

# 4. Venn diagram: shared and unique OTUs

# ----------------------------

# Convert abundance table to binary (presence/absence)

otu_binary <- otu_data

otu_binary[otu_binary > 0] <- 1

# Extract sample groups

group_H <- rownames(sample_info[sample_info$Group == "Healthy", ])

group_HCC <- rownames(sample_info[sample_info$Group == "Hepatocellular carcinoma", ])

# Get OTUs present in each group

otu_H <- rownames(otu_binary[, colnames(otu_binary) %in% group_H][rowSums(otu_binary[, colnames(otu_binary) %in% group_H]) > 0, ])

otu_HCC <- rownames(otu_binary[, colnames(otu_binary) %in% group_HCC][rowSums(otu_binary[, colnames(otu_binary) %in% group_HCC]) > 0, ])

# Draw Venn diagram

venn_plot <- venn.diagram(

x = list(Healthy = otu_H, HCC = otu_HCC),

filename = NULL,

col = "transparent",

fill = c("dodgerblue", "goldenrod1"),

alpha = 0.5,

label.col = "black",

cex = 1.5,

fontfamily = "Helvetica",

cat.col = c("dodgerblue", "goldenrod1"),

cat.cex = 1.2,

cat.fontfamily = "Helvetica"

)

grid.newpage()

grid.draw(venn_plot)

# ----------------------------

# 5. Taxonomic composition at Phylum level

# ----------------------------

# Read taxonomy annotation

taxa_data <- read.table("result/taxonomy_table.txt", header = TRUE, row.names = 1, sep = "\t")

tax_table <- tax_table(as.matrix(taxa_data))

# Merge with phyloseq object

physeq_all <- merge_phyloseq(physeq, tax_table)

# Collapse to Phylum level

physeq_phylum <- tax_glom(physeq_all, taxrank = "Phylum")

# Transform to relative abundance

physeq_rel <- transform_sample_counts(physeq_phylum, function(x) x / sum(x))

# Plot stacked barplot

barplot_phylum <- plot_bar(physeq_rel, fill = "Phylum") +

theme_bw() +

facet_wrap(~Group, scales = "free_x") +

scale_fill_brewer(palette = "Set3") +

labs(title = "Microbial Composition at Phylum Level", y = "Relative Abundance")

# ----------------------------

# 6. Save output figures

# ----------------------------

ggsave("Figure_Alpha_Shannon.png", p_alpha, width = 6, height = 5)

ggsave("Figure_Beta_PCoA.png", p_beta, width = 6, height = 5)

ggsave("Figure_Barplot_Phylum.png", barplot_phylum, width = 10, height = 5)
