# Supplementary material for: Tumor-associated bacteria activate PRDX1-driven glycolysis to promote immune evasion and PD-1 antibody resistance in hepatocellular carcinoma
Source: Front Microbiol. 2025 Jul 7;16:1599691. doi: 10.3389/fmicb.2025.1599691 (PMC12277338; doi:10.3389/fmicb.2025.1599691)
Supplement: Supplementary file 9 [file Data_Sheet_2.docx]

Supplementary Code 2

Steps:

1. Data loading and sample merging (7 H5-format files including HCC and Normal samples)
2. Quality control (filtering of low-quality cells)
3. Data normalization, PCA, and batch correction using Harmony
4. Clustering and dimensionality reduction with UMAP visualization
5. Automatic cell type annotation using the SingleR package
6. Quantification of NK cell proportion changes
7. Differential expression analysis on hepatocytes (including genes such as PRDX1)
8. Saving the final annotated Seurat object

Script:

# Load required libraries

library(Seurat)

library(ggplot2)

library(cowplot)

library(Matrix)

library(dplyr)

library(ggsci)

setwd("your/working/directory") # Set working directory

#### 1. Load sample data (H5 format) ####

# Read 10X HDF5 format files for HCC and Normal samples

HCC1 <- Read10X_h5("GSM5709317_1HT1_raw_feature_bc_matrix.h5")

HCC2 <- Read10X_h5("GSM5709326_3HT2_raw_feature_bc_matrix.h5")

HCC3 <- Read10X_h5("GSM5709327_3HT3_raw_feature_bc_matrix.h5")

HCC4 <- Read10X_h5("GSM5709336_2HT1_raw_feature_bc_matrix.h5")

Normal1 <- Read10X_h5("GSM5709316_1HN_raw_feature_bc_matrix.h5")

Normal2 <- Read10X_h5("GSM5709324_3HN_raw_feature_bc_matrix.h5")

Normal3 <- Read10X_h5("GSM5709329_4HN_raw_feature_bc_matrix.h5")

#### 2. Create Seurat objects ####

# Create Seurat objects for each sample with filtering

HCC1 <- CreateSeuratObject(HCC1, project = "HCC1", min.cells = 10, min.features = 200)

HCC1$treatment <- "HCC"

HCC2 <- CreateSeuratObject(HCC2, project = "HCC2", min.cells = 10, min.features = 200)

HCC2$treatment <- "HCC"

HCC3 <- CreateSeuratObject(HCC3, project = "HCC3", min.cells = 10, min.features = 200)

HCC3$treatment <- "HCC"

HCC4 <- CreateSeuratObject(HCC4, project = "HCC4", min.cells = 10, min.features = 200)

HCC4$treatment <- "HCC"

Normal1 <- CreateSeuratObject(Normal1, project = "Normal1", min.cells = 10, min.features = 200)

Normal1$treatment <- "Normal"

Normal2 <- CreateSeuratObject(Normal2, project = "Normal2", min.cells = 10, min.features = 200)

Normal2$treatment <- "Normal"

Normal3 <- CreateSeuratObject(Normal3, project = "Normal3", min.cells = 10, min.features = 200)

Normal3$treatment <- "Normal"

#### 3. Merge all samples into one Seurat object ####

ScRNA <- merge(HCC1, y = c(HCC2, HCC3, HCC4, Normal1, Normal2, Normal3),

add.cell.ids = c("HCC1", "HCC2", "HCC3", "HCC4", "Normal1", "Normal2", "Normal3"))

saveRDS(ScRNA, file = "./ScRNA_merge.rds") # Save merged object

#### 4. Quality control ####

# Calculate mitochondrial gene percentage

ScRNA[["percent.mt"]] <- PercentageFeatureSet(ScRNA, pattern = "^MT-")

# Visualize QC metrics

VlnPlot(ScRNA, features = c("nFeature_RNA", "nCount_RNA", "percent.mt"), ncol = 3,

group.by = "orig.ident", pt.size = 0)

# Scatter plots for QC relationships

plot1 <- FeatureScatter(ScRNA, feature1 = "nCount_RNA", feature2 = "percent.mt")

plot2 <- FeatureScatter(ScRNA, feature1 = "nCount_RNA", feature2 = "nFeature_RNA")

# Save QC plots to PDF

pdf("QC-FeatureScatter.pdf", width = 8, height = 4.5)

CombinePlots(plots = list(plot1, plot2), legend = "right")

dev.off()

# Filter cells based on QC thresholds

ScRNA <- subset(ScRNA, subset = nFeature_RNA > 200 & nFeature_RNA < 5000 & percent.mt < 20)

#### 5. Normalize data and identify variable features ####

ScRNA <- NormalizeData(ScRNA, normalization.method = "LogNormalize", scale.factor = 10000)

ScRNA <- FindVariableFeatures(ScRNA, selection.method = "vst", nfeatures = 2000)

# Plot top variable genes

top10 <- head(VariableFeatures(ScRNA), 10)

plot1 <- VariableFeaturePlot(ScRNA)

plot2 <- LabelPoints(plot = plot1, points = top10, repel = TRUE, size = 2.5)

CombinePlots(plots = list(plot1, plot2), legend = "bottom")

#### 6. Cell cycle scoring ####

s.genes <- cc.genes$s.genes

g2m.genes <- cc.genes$g2m.genes

ScRNA <- CellCycleScoring(ScRNA, s.features = s.genes, g2m.features = g2m.genes, set.ident = TRUE)

VlnPlot(ScRNA, features = c("S.Score", "G2M.Score"), group.by = "treatment", pt.size = 0)

#### 7. Data scaling and PCA ####

ScRNA <- ScaleData(ScRNA, vars.to.regress = c("percent.mt", "orig.ident", "S.Score", "G2M.Score"))

ScRNA <- RunPCA(ScRNA, npcs = 50)

ElbowPlot(ScRNA, ndims = 50)

#### 8. Batch effect correction using Harmony ####

library(harmony)

ScRNA <- RunHarmony(ScRNA, group.by.vars = "orig.ident", plot_convergence = TRUE)

#### 9. Clustering and dimensionality reduction ####

ScRNA <- ScRNA %>%

RunUMAP(reduction = "harmony", dims = 1:30) %>%

RunTSNE(reduction = "harmony", dims = 1:30) %>%

FindNeighbors(reduction = "harmony", dims = 1:30)

ScRNA <- FindClusters(ScRNA, resolution = seq(from = 0.1, to = 1.0, by = 0.1))

library(clustree)

clustree(ScRNA)

Idents(ScRNA) <- "RNA_snn_res.1"

ScRNA$seurat_clusters <- ScRNA@active.ident

# Visualize clustering by tSNE/UMAP

DimPlot(ScRNA, reduction = "tsne", label = TRUE)

DimPlot(ScRNA, reduction = "umap", label = TRUE)

#### 10. Annotate and identify marker genes ####

ScRNA.markers <- FindAllMarkers(ScRNA, only.pos = TRUE, min.pct = 0.25, logfc.threshold = 0.25)

write.csv(ScRNA.markers, "ScRNA.all.markers.csv")

#### 11. Cell type annotation using SingleR ####

library(SingleR)

library(celldex)

refdata <- HumanPrimaryCellAtlasData()

testdata <- GetAssayData(ScRNA, slot = "data")

clusters <- ScRNA@meta.data$seurat_clusters

cellpred <- SingleR(test = testdata, ref = refdata, labels = refdata$label.main,

method = "cluster", clusters = clusters,

assay.type.test = "logcounts", assay.type.ref = "logcounts")

# Map predicted cell types back to Seurat object

celltype <- data.frame(ClusterID = rownames(cellpred), celltype = cellpred$labels, stringsAsFactors = FALSE)

ScRNA@meta.data$celltype <- "Unknown"

for (i in 1:nrow(celltype)) {

ScRNA@meta.data[which(ScRNA@meta.data$seurat_clusters == celltype$ClusterID[i]), 'celltype'] <- celltype$celltype[i]

}

# UMAP plot split by treatment and colored by cell type

DimPlot(ScRNA, group.by = "celltype", label = TRUE, reduction = "umap", split.by = "treatment")

save(ScRNA, file = "ScRNA_annotated.RData")

show_annotation_name = FALSE,

gp = gpar(col = 'black'),

col = list(CellType = c('T cells' = "#e9148f",

'B cells' = "#F6F5B4",

'NK cells' = "#e5192c",

'TAMs' = "mediumturquoise",

'pDCs' = "#3a77b7",

'Hepatocytes' = "#F3B1A0",

'Plasma cells' = "#813c93",

'Mast cells' = "#F7A24F",

'Granulocytes' = "#0E8585",

'Fibroblasts' = "#0787C3",

'Endothelial cells' = "#F9DF91",

'Epithelial cells' = "#FFB300",

'Megakaryocytes' = "#F08E64")))

# Draw heatmap

Heatmap(marker_exp,

cluster_rows = FALSE,

cluster_columns = FALSE,

show_column_names = FALSE,

show_row_names = TRUE,

column_title = NULL,

heatmap_legend_param = list(title = 'Expression Level'),

col = colorRampPalette(c("#C5DEBA", "mediumturquoise", "#e5192c"))(100),

border = TRUE,

rect_gp = gpar(col = "black", lwd = 1),

row_names_gp = gpar(fontsize = 10),

column_names_gp = gpar(fontsize = 10),

top_annotation = top_anno)

library(Seurat)

library(dplyr)

library(future)

library(future.apply)

library(stringr)

#devtools::install_github("sqjin/NMF")##cell-cell interaction

library(CellChat)

normaldata<-ScRNA1[,ScRNA1$treatment=="Normal"]

tumordata<-ScRNA1[,ScRNA1$treatment=="HCC"]

normal.input <- GetAssayData(normaldata, assay = "RNA", slot = "data") # normalized data matrix

labels <- factor(normaldata$celltype,levels=levels(Idents(ScRNA1)))

labels

meta <- data.frame(group = labels, row.names = rownames(normaldata@meta.data))

cellchat_normal <- createCellChat(object = normal.input, meta = meta, group.by = "group")

saveRDS(cellchat_normal,file="./normalcellchat_1.rds")

tumor.input <- GetAssayData(tumordata, assay = "RNA", slot = "data") # normalized data matrix

labels <- factor(tumordata$celltype,levels=levels(Idents(ScRNA1)))

meta <- data.frame(group = labels, row.names = rownames(tumordata@meta.data))

cellchat_tumor <- createCellChat(object = tumor.input, meta = meta, group.by = "group")

saveRDS(cellchat_tumor,file="./tumorcellchat_1.rds")

normal_cellchat<-cellchat_normal

tumor_cellchat<-readRDS(file="./tumorcellchat_1.rds")

normal_cellchat@DB <- CellChatDB.human

normal_cellchat <- subsetData(normal_cellchat)

# subset the expression data of signaling genes for saving computation cost

#future::plan("multiprocess", workers = 2) # do parallel

#> Warning: [ONE-TIME WARNING] Forked processing ('multicore') is disabled

#> in future (>= 1.13.0) when running R from RStudio, because it is

#> considered unstable. Because of this, plan("multicore") will fall

#> back to plan("sequential"), and plan("multiprocess") will fall back to

#> plan("multisession") - not plan("multicore") as in the past. For more details,

#> how to control forked processing or not, and how to silence this warning in

#> future R sessions, see ?future::supportsMulticore

normal_cellchat <- identifyOverExpressedGenes(normal_cellchat)

normal_cellchat <- identifyOverExpressedInteractions(normal_cellchat)

normal_cellchat <- projectData(normal_cellchat, PPI.human)

normal_cellchat <- computeCommunProb(normal_cellchat, raw.use = TRUE)

# Filter out the cell-cell communication if there are only few number of cells in certain cell groups

normal_cellchat <- filterCommunication(normal_cellchat, min.cells = 1)

normal_cellchat <- computeCommunProbPathway(normal_cellchat)

normal_cellchat <- netAnalysis_computeCentrality(normal_cellchat, slot.name = "netP")

normal_cellchat <- aggregateNet(normal_cellchat)

groupSize <- as.numeric(table(normal_cellchat@idents))

groupSize

table(normal_cellchat@idents)

par(mfrow = c(1,2), xpd=TRUE)

netVisual_circle(normal_cellchat@net$count,arrow.size = 0.01, vertex.weight = groupSize, weight.scale = T, label.edge= F, title.name = "Number of interactions")

netVisual_circle(normal_cellchat@net$weight, arrow.size = 0.01,vertex.weight = groupSize, weight.scale = T, label.edge= F, title.name = "Interaction weights/strength")

mat <- normal_cellchat@net$weight

par(mfrow = c(2,3), xpd=TRUE) #Set plotting panel: 2 rows × 3 columns

for (i in 1:nrow(mat)) {

mat2 <- matrix(0, nrow = nrow(mat), ncol = ncol(mat), dimnames = dimnames(mat))

mat2[i, ] <- mat[i, ]

netVisual_circle(mat2, vertex.weight = groupSize, weight.scale = T, edge.weight.max = max(mat), title.name = rownames(mat)[i])

}

tumor_cellchat<-readRDS(file="./tumorcellchat_1.rds")

tumor_cellchat@DB <- CellChatDB.human

#gc()

tumor_cellchat <- subsetData(tumor_cellchat) # subset the expression data of signaling genes for saving computation cost

tumor_cellchat <- identifyOverExpressedGenes(tumor_cellchat)

tumor_cellchat <- identifyOverExpressedInteractions(tumor_cellchat)

tumor_cellchat <- projectData(tumor_cellchat, PPI.human)

tumor_cellchat <- computeCommunProb(tumor_cellchat, raw.use = TRUE)

# Filter out the cell-cell communication if there are only few number of cells in certain cell groups

tumor_cellchat <- filterCommunication(tumor_cellchat, min.cells = 1)

tumor_cellchat <- computeCommunProbPathway(tumor_cellchat)

tumor_cellchat <- netAnalysis_computeCentrality(tumor_cellchat, slot.name = "netP")

tumor_cellchat <- aggregateNet(tumor_cellchat)

par(mfrow = c(2,2), xpd=TRUE)

groupSize <- as.numeric(table(tumor_cellchat@idents))

groupSize

table(tumor_cellchat@idents)

par(mfrow = c(1,2), xpd=TRUE)

netVisual_circle(tumor_cellchat@net$count,arrow.size = 0.01, vertex.weight = groupSize, weight.scale = T, label.edge= F, title.name = "Number of interactions")

netVisual_circle(tumor_cellchat@net$weight, arrow.size = 0.01,vertex.weight = groupSize, weight.scale = T, label.edge= F, title.name = "Interaction weights/strength")

mat <- tumor_cellchat@net$weight

par(mfrow = c(2,3), xpd=TRUE) #Set plotting panel: 2 rows × 3 columns

for (i in 1:nrow(mat)) {

mat2 <- matrix(0, nrow = nrow(mat), ncol = ncol(mat), dimnames = dimnames(mat))

mat2[i, ] <- mat[i, ]

netVisual_circle(mat2, vertex.weight = groupSize, weight.scale = T, edge.weight.max = max(mat), title.name = rownames(mat)[i])

}

####8.Differential expression analysis####

Ecs <- subset(ScRNA1, celltype=="Hepatocytes")#Subset hepatocytes from annotated Seurat object

diff_Ecs <- FindMarkers(Ecs, min.pct = 0.25,

logfc.threshold = 0,

group.by = "treatment",

ident.1 ="HCC",

ident.2="Normal")

write.csv(diff_Ecs,"./diff_Ecs-0.csv")

diff_Ecs <- FindMarkers(Ecs, min.pct = 0.25,

logfc.threshold = 0.5,

group.by = "treatment",

ident.1 ="HCC",

ident.2="Normal")

write.csv(diff_Ecs,"./diff_Ecs-0.5.csv")

#Visualize expression of canonical marker genes across UMAP

FeaturePlot(ScRNA,features = c("SENP3"),reduction = "umap",label = T,split.by = "treatment")

FeaturePlot(ScRNA1,features = c("LGALS3","LGALS4","CA1","CA2"),reduction = "umap",label = F)

#COL1A2，BGN，ACTA2

FeaturePlot(ScRNA1,features = c("CD3E","CD79A","EPCAM","KDR","CD68","COL1A1","CMA1"),reduction = "umap",label = F)
